# An *In-Silico* Model of Lipoprotein Metabolism and Kinetics for the Evaluation of Targets and Biomarkers in the Reverse Cholesterol Transport Pathway

**DOI:** 10.1371/journal.pcbi.1003509

**Published:** 2014-03-13

**Authors:** James Lu, Katrin Hübner, M. Nazeem Nanjee, Eliot A. Brinton, Norman A. Mazer

**Affiliations:** 1 F. Hoffmann-La Roche AG, pRED, Pharma Research & Early Development, Clinical Pharmacology, Basel, Switzerland; 2 BioQuant, University of Heidelberg, Heidelberg, Germany; 3 Division of Cardiovascular Genetics, University of Utah, Salt Lake City, Utah, United States of America; 4 Utah Foundation for Biomedical Research, Salt Lake City, Utah, United States of America; University of Michigan, United States of America

## Abstract

High-density lipoprotein (HDL) is believed to play an important role in lowering cardiovascular disease (CVD) risk by mediating the process of reverse cholesterol transport (RCT). Via RCT, excess cholesterol from peripheral tissues is carried back to the liver and hence should lead to the reduction of atherosclerotic plaques. The recent failures of HDL-cholesterol (HDL-C) raising therapies have initiated a re-examination of the link between CVD risk and the rate of RCT, and have brought into question whether all target modulations that raise HDL-C would be atheroprotective. To help address these issues, a novel *in-silico* model has been built to incorporate modern concepts of HDL biology, including: the geometric structure of HDL linking the core radius with the number of ApoA-I molecules on it, and the regeneration of lipid-poor ApoA-I from spherical HDL due to remodeling processes. The ODE model has been calibrated using data from the literature and validated by simulating additional experiments not used in the calibration. Using a virtual population, we show that the model provides possible explanations for a number of well-known relationships in cholesterol metabolism, including the epidemiological relationship between HDL-C and CVD risk and the correlations between some HDL-related lipoprotein markers. In particular, the model has been used to explore two HDL-C raising target modulations, Cholesteryl Ester Transfer Protein (CETP) inhibition and ATP-binding cassette transporter member 1 (ABCA1) up-regulation. It predicts that while CETP inhibition would not result in an increased RCT rate, ABCA1 up-regulation should increase both HDL-C and RCT rate. Furthermore, the model predicts the two target modulations result in distinct changes in the lipoprotein measures. Finally, the model also allows for an evaluation of two candidate biomarkers for *in-vivo* whole-body ABCA1 activity: the absolute concentration and the % lipid-poor ApoA-I. These findings illustrate the potential utility of the model in drug development.

## Author Summary

Epidemiological studies have shown a strong inverse association between HDL-C and cardiovascular risk and led to the formulation of the “HDL cholesterol hypothesis”: under this hypothesis, interventions raising HDL-C should decrease risk. However, the recent failures of HDL-C raising therapies in improving cardiovascular disease risk in outcomes trials have suggested a need to revise the hypothesis to account for the contrary data. An “HDL flux hypothesis” has emerged: it is not HDL-C level *per se* which forms the basis for reducing risk, but it is the flux rate of reverse cholesterol transport that drives risk reduction. We propose that, the concentration of HDL cholesteryl ester in plasma simply reflects the ratio of input rate of reverse cholesterol transport into the HDL compartments to its clearance rate. A challenge in identifying targets under the new conceptual framework is the feedback process that occurs between the input rate and the clearance rate of HDL-C. To meet this challenge, we have built a systems model which incorporates the main processes of HDL metabolism to elucidate the relationships between target modulations and the reverse cholesterol transport rate.

## Introduction

Epidemiological studies have shown that high levels of low-density lipoprotein cholesterol (LDL-C) as well as low levels of high-density lipoprotein cholesterol (HDL-C) are associated with increased cardiovascular disease (CVD) risk [1], [2]. While LDL-C lowering therapies have been shown consistently to reduce CVD risk, there is significant residual risk that remains to be managed [2]. The strong inverse association between HDL-C and CVD risk has led to the “HDL-C hypothesis”, whereby all HDL-C raising therapies should be anti-atherogenic [2], [3]. Currently, the anti-atherogenic activity of HDL is mainly attributed to its role in mediating reverse cholesterol transport (RCT), whereby cholesterol is effluxed from peripheral tissues and transported to the liver for biliary excretion [4]. However, the recent failures of a number of HDL-C raising intervention trials [5]–[7] have called for a re-examination of the HDL-C hypothesis. It has long been thought that HDL-C is a reliable biomarker for cholesterol efflux from tissues [8]. However, the several recent failed HDL-C raising intervention trials provide mounting evidence that at least under certain conditions, the plasma concentration of HDL-C, a very simple and static measure, is inadequate for characterizing the rate of RCT, which is a complex and dynamic process [8]. A revision of the HDL-C hypothesis to the “HDL flux hypothesis” has been proposed, whereby interventions should be aimed at promoting cholesterol efflux to HDL, and hence the overall RCT rate, independently of their effects on HDL-C levels [9], [10]. Hence, there is now a pressing need to better understand the role of HDL-C raising targets in the context of RCT and to identify biomarkers which could provide information on the flux rate through the RCT pathway [8]. Our modeling effort is focused on addressing these issues.

A number of previous mathematical models have focused on various aspects of lipid metabolism; see [11], [12] for recent reviews. Of the existing models, some describe metabolic processes at a mechanistic level [13]–[19], while others have been empirically derived from tracer kinetic studies [20]–[22]. In general these models were built to describe the dynamics of HDL and the other major lipoprotein classes, which include LDL, intermediate density lipoprotein (IDL) and very low density lipoprotein (VLDL), describing lipid transport between these particles mediated by the cholesteryl ester transport protein (CETP) in the normal or basal state, and the effects of genetic mutations and/or drug interventions on these processes. While valuable insights have been gained from these models, none can be used to predict the associated changes in the RCT rate since they lack a mechanistic description of ApoA-I dynamics and other key processes involved in the RCT pathway. The latter include the lipidation of lipid-poor ApoA-I via its interaction with ATP-binding cassette transporter member 1 (ABCA1), the key process in the initiation of RCT [8], as well as processes of HDL remodeling which lead to the delivery of cholesterol from HDL to other lipoproteins and cells, and the regeneration of lipid poor ApoA-I [23]. In all the existing models except the ones by Hübner *et al* [16] and Adiels *et al* [24], the dynamics of apolipoproteins that cover the surface of lipoprotein particles are not described. While each VLDL, IDL and LDL particle contains only one ApoB molecule per particle, for HDL particles the number of ApoA-I molecules per particle may vary from 2 to 4 or more depending on HDL size [25]. This variation results from HDL remodeling processes such as particle fusion, CETP-mediated lipid transport, lipolysis and esterification whereby particles can gain or lose core lipid content as well as ApoA-I molecules [23]. While it has been shown experimentally and theoretically that the number of ApoA-I molecules on a given HDL particle is intrinsically linked to the particle size [25], this important relationship has yet to be incorporated into a mechanistic model of HDL metabolism.

In this paper, we propose a novel model of lipoprotein metabolism and kinetics (the LMK model) that provides an integrated description of the dynamics of cholesterol and ApoA-I in plasma. In particular, the model captures the initiation of RCT from the lipidation of lipid-poor ApoA-I by the ABCA1 transporter, the generation of nascent discoidal and nascent spherical particles, HDL particle fusion, CETP mediated lipid transfer between HDL and other lipoproteins, and the dissociation of excess ApoA-I from mature spherical *α*-HDL due to remodeling processes. The model is calibrated to: lipoprotein measures for normal and CETP deficient subjects; cholesteryl ester (CE) and ApoA-I fluxes measured in normal subjects; data on the fractional catabolic rate (FCR) of ApoA-I. The structure and the kinetic constants of our model provide an explanation for the relationship between FCR of ApoA-I and HDL particle size. To our knowledge the LMK model is the first to provide a mechanistic basis for the linkage between the metabolism of ApoA-I and the cholesterol component of HDL. The model has been validated by simulating patients with genetic mutations in the HDL metabolism pathway and the predictions are compared with lipoprotein measures reported in literature. Finally, the model was used to evaluate targets that could potentially increase RCT and to identify relevant biomarkers, as part of the effort to support drug discovery and development using a model-based approach.

## Results/Discussion

### Model structure

**Figure 1:**
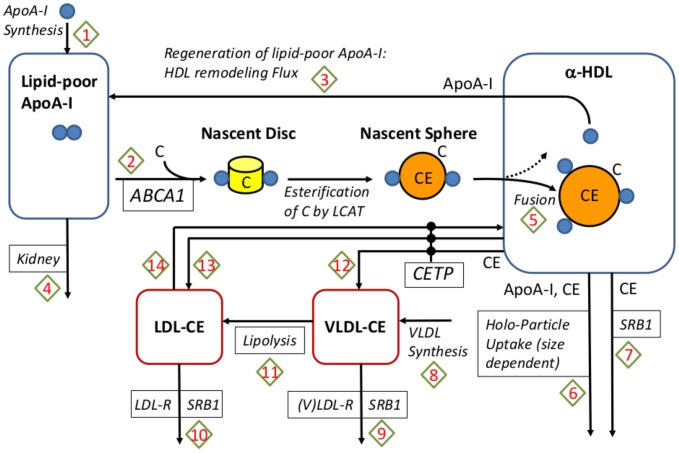
A schematic representation of the model. The arrows shown in the diagram denote the processes represented by the model and the boxes with italicized text denote mediators that are explicitly represented. The process arrows are numbered, refering to the reaction number shown in Table 2. The arrows leading from the nascent sphere towards the *α*-HDL pool represent the 2 scenarios that may occur in the transformation of newly formed particles: they may either enter the *α*-HDL pool as distinct particles (the dashed arrow) or fuse with the existing ones (solid arrow).

The LMK model is shown schematically in Figure 1, focused on the RCT pathway and a number of targets contained within it, for instance CETP, ABCA1, ApoA-I and SRB1. The LMK model describes the synthesis of ApoA-I and the initiation of RCT by the interaction of lipid-poor ApoA-I with ABCA1 leading to the formation of mature, spherical *α*-HDL. The HDL remodeling processes represented in the model include: the fusion of spherical HDL particles (arrow 5 of Figure 1); the exchange and elimination of CE in spherical HDL by interaction with CETP (arrows 12–14) and SRB1 (arrow 7); the regeneration of lipid-poor ApoA-I from spherical HDL particles (arrow 3). Lipid-poor ApoA-I is assumed to be eliminated via the kidney (arrow 4), while the spherical HDL particles are assumed to be eliminated by a holo-uptake mechanism with a rate dependent on the particle size (arrow 6). The transfer and elimination of CE in LDL and VLDL pools are also represented (arrows 9–11). Our approach is to adequately describe the metabolic processes, while keeping the model as simple as possible. The representations of lipoprotein components and metabolic processes in the LMK model reflect these principles.

#### Lipoprotein representation

While HDL particles are heterogeneous in size and composition [8], for the purposes of understanding RCT we only consider two HDL particle classes: spherical, *α*-HDL and small, lipid-poor ApoA-I. Amongst the apolipoproteins and lipid species contained in *α*-HDL particles, the LMK model has an explicit representation of ApoA-I and CE. Although there are a large number of species in the HDL proteome (e.g., ApoA-II, ApoE) and lipidome (e.g., triglycerides, phospholipids) which may be relevant in particular diseased states, they play a secondary role in the characterization of RCT. We make the assumption that the protein moiety contains 60% ApoA-I by weight, with all other proteins contributing the remaining 40%. This is within the range of values reported in literature [25], [26]. Under this assumption, the total concentration of ApoA-I is represented as an explicit variable that changes as a direct result of the metabolic processes described in the model while ApoA-II and other HDL apolipoproteins are implicit quantities: namely, they are assumed to change in concert with ApoA-I so as to keep the weight fraction constant. Similarly, the LMK model explicitly represents CE in the particle classes of *α*-HDL, VLDL and LDL but represents TG in *α*-HDL only implicitly. That is, the ratio of TG/CE in *α*-HDL particles is assumed to be 13% which is consistent with the range of values reported in healthy subjects [25], [27]. The amounts of free cholesterol (FC) and phospholipids (PL) per HDL particle are implicitly represented in the LMK model: they depend on the *α*-HDL size, in a manner analogous to the treatment of PL in [16]. In particular, given the CE content of an *α*-HDL particle its core size can be inferred and the FC and PL content on the surface can be computed using the updated Shen model; see [25]. With our choice of lipoprotein representation, the species represented in the model are given in Table 1.

**Table 1:** Species represented in the model.

Symbol	Description	Units
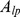	Lipid-poor ApoA-I	mg/dL
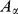	ApoA-I in the *α*-HDL pool	mg/dL
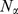	Particle concentration of *α*-HDL	mmol/dL
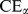	cholesteryl ester in *α*-HDL	mg/dL
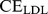	cholesteryl ester in LDL	mg/dL
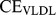	cholesteryl ester in VLDL	mg/dL

#### Metabolic processes

The full list of reactions represented in the LMK model (as schematized in Figure 1) is shown in Table 2. We would like to point out that the remodeling flux (arrow 3 of Figure 1) based on geometric concepts developed in [25] is an original contribution of our work. The remodeling flux expression, 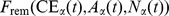, represents the excess ApoA-I within the pool of *α*-HDL particles given the core cholesteryl ester content, particle concentration and the amount of ApoA-I covering the surface. Its derivation based on geometric concepts of *α*-HDL particles is discussed in more detail in the Methods section. The holo-uptake of *α*-HDL particles is thought to be mediated by a number of receptors, which are not well understood [28], [29]. In order to account for the possible size-dependence in the uptake rate of *α*-HDL particles, the functional dependence 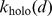 is utilized. This is also discussed in more detail in the Methods section.

**Table 2:** Reactions represented in the model.

#	Reaction	Description	Rate expression	Ref.
1	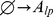	ApoA-I synthesis	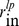	[22], [88]
2	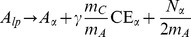	Initiation of RCT by interaction with ABCA1	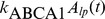	[79], [80], [89]
3	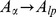	Regeneration of lipid-poor ApoA-I via HDL remodeling	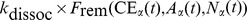	[22], [25], [76], [90]–[92]
4	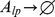	Kidney removal of lipid-poor ApoA-I	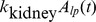	[43], [48]
5	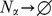	Fusion of nascent spherical particles with mature *α*-HDL	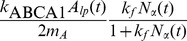	[23], [93]
6	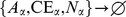	HDL particle holo-uptake	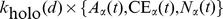	[28], [29]
7	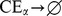	SR-B1 mediated removal of CE from HDL particles	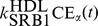	[4], [8], [18], [36]
8	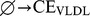	Synthesis of CE in VLDL	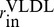	[20], [21]
9	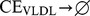	Elimination of CE from VLDL	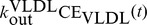	[20], [21]
10	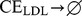	Elimination CE from LDL	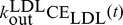	[20], [21]
11	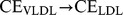	VLDL conversion to LDL via lipolysis	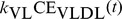	[20]
12	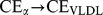	CETP mediated CE transfer from HDL to VLDL	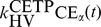	[13], [17], [18], [20], [30]
13	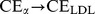	CETP mediated CE transfer from HDL to LDL	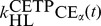	[13], [20], [30]
14	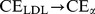	CETP mediated CE transfer from LDL to HDL	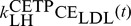	[13], [18], [20], [30]

The model constants are shown in Table 3 while the list of parameters are given in Table 4; the prior values of parameters and their posterior estimates are discussed in the next section. With the list of reactions given in Table 2, the LMK model can be expressed as the following system of ODEs:
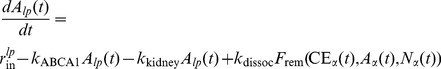
(1a)

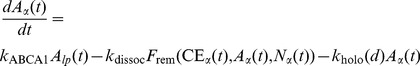
(1b)

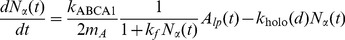
(1c)

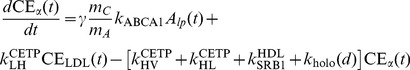
(1d)

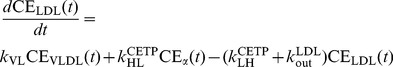
(1e)

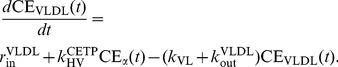
(1f)One important quantity that the LMK model can help to assess is the rate of *reverse cholesterol transport* (RCT) at the whole-body level. This quantity is thought to play an important role in determining cardiovascular disease risk, but is experimentally challenging to assess. Using the LMK model, we are able to quantify the flux rate of free cholesterol into the nascent disc particles mediated by ABCA1: in particular, this is given by
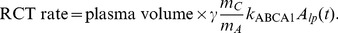
(2)The term 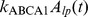 represents the transformation rate of lipid-poor ApoA-I to nascent discs, which subsequently enter the *α*-HDL pool. The parameter *γ* describes the number of cholesterol molecules per ApoA-I in the nascent discs. 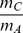 converts the molecular mass of ApoA-I to cholesterol. Finally, the volume of plasma converts RCT rate to the whole-body level: we assume that plasma volume = 3.15 L in a 70 kg adult [22]. As illustrated in Figure 1, nascent discs are transformed into nascent spheres (as mediated by the LCAT enzyme [30]) which are assumed to have in their cores *γ* CE molecules per ApoA-I. Hence, the RCT expression (2) also represents the input rate of HDL-CE into the plasma *α*-HDL pool. Note that the factor 2 in the expression for reaction 2 (initiation of RCT) accounts for the assumption that there are 2 ApoA-I molecules per nascent HDL particle.

**Table 3:** Model constants.

Constant	Description	Unit	Value	Ref.
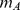	Molecular weight of ApoA-I	g/mol	28500	[16]
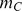	Molecular weight of cholesterol (free and esterified)	g/mol	386	[16]
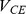	Molecular volume of cholesteryl ester	Å^3^	1068	[25]
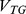	Molecular volume of triglyceride	Å^3^	1556	[25]
*t*	Thickness of HDL surface	Å	20.2	[25]

By convention, the cholesteryl ester mass is measured by quantifying the equivalent mass of free cholesterol.

**Table 4:** Model parameters.

	Description	Unit
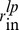	Synthesis rate of ApoA-I	mg/dL/day
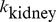	Rate of kidney elimination	pool/day
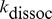	Dissociation rate of excess ApoA-I	pool/day
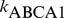	Rate constant in the lipidation of lipid-poor ApoA-I via ABCA1	pool/day
*γ*	Stoichiometry of FC to ApoA-I in nascent discs	unitless
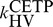	Rate constant of CE transfer: HDL to VLDL	pool/day
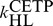	Rate constant of CE transfer: HDL to LDL	pool/day
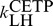	Rate constant of CE transfer: LDL to HDL	pool/day
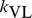	Rate constant of CE transfer: VLDL to LDL	pool/day
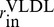	Synthesis rate of CE to VLDL	mg/dL/day
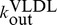	Rate constant of CE elimination from VLDL	pool/day
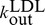	Rate constant of CE elimination from LDL	pool/day
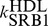	Rate constant of SRB1-mediated CE elimination from HDL	pool/day
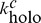	Constant contribution to the rate of *α*-HDL holo-particle uptake	pool/day
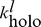	Size-dependent contribution to the rate of *α*-HDL holo-particle uptake	pool/day/nm
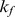	Parameter governing the particle concentration dependence of fusion rate	1/(mmol/dL)

### Model calibration

#### Parameter estimates: Prior and posterior

The Bayesian approach for parameter estimation is a well established methodology which has found applications in various fields of science [31], including parameter estimation for models of cellular processes [32], [33] as well as pharmacokinetics and pharmacodynamics (PK/PD) [34], [35]. Under this framework, it is assumed that a prior distribution is available for (some) parameters as a result of previous experimental studies. In combination with calibration data, the posterior distribution for the parameters is obtained.

For most of the LMK model parameters, prior estimates are available from literature studies; a detailed discussion of the references from which parameter estimates and their uncertainties are obtained is given in the Methods section. Using the model calibration procedure as discussed in the Methods section, the prior is combined with calibration data to give rise to the posterior estimates. A list of the prior and posterior values of parameters is given in Table 5. It is worthwhile noting that, for the most part, the *maximum a posteriori* (MAP) estimate obtained by the calibration process does not depart significantly from the prior. This indicates that the calibration data are fairly consistent with the prior estimates. One exception is the parameter 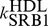, which is increased significantly from its prior beyond the 1 SD value. This result is in agreement with experimental evidence that SRB1 plays a significant role in mediating HDL-CE removal from HDL particles [36], in contrast to the expectation of a previous tracer kinetics study [21]. The discrepancy may be attributed to the limitation of tracer kinetics studies (for instance, [21]) to be able to fully identify the SRB1 contribution. Finally, it can be seen that the calibration data are sufficiently informative to allow relatively precise estimates for the parameters 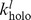 and 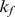, for which there was no prior information. It is worth noting the negative sign in the estimate for 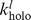, which implies that the *α*-HDL holo-particle uptake rate decreases with particle size. The sign of this size-dependence is consistent with the hepatic endocytic receptor (mitochondrial ATP synthase subunit *β*) having a higher affinity for the (smaller) HDL-3 as compared to the (larger) HDL-2 [29], [37], [38].

**Table 5:** Prior and posterior estimates of model parameters corresponding to the “nominal subject.”

Parameter	Prior (mean±SD)	Posterior (mean±SD)
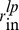	27.44±1.18	28.46±1.13
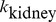	5.19±2.60	2.42±0.78
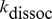	174±312	170±191
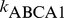	96.24±17.55	95.18±15.73
*γ*	7.55±3.94	10.17±2.19
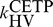	1.47±0.58	1.49±0.24
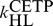	5.47±2.05	6.92±0.81
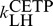	1.98±0.70	2.89±0.34
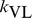	7.52±0.94	7.70±0.84
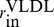	0.96±0.46	1.50±0.45
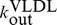	0.88±0.37	1.30±0.35
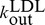	0.67±0.08	0.64±0.07
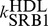	0.31±0.12	0.60±0.08
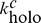	0.14±0.026	0.13±0.022
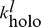	0	−0.016±0.004
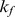	0	5000±1544

The prior parameter values were estimated from literature as discussed under the Methods section. The SDs of the posterior distribution were estimated using the Fisher Information Matrix.

#### Calibration data and model explanatory power

In order to identify parameter values using the Bayesian approach, calibration data are needed. However, the choice of calibration data should be made not only for the purpose of quantifying parameters, but also with the consideration for the potential utility and the explanatory power of the model. More specifically, choosing the right types of calibration data can help to increase confidence in a model's predictions of specific scenarios; in addition, model calibration is also an opportunity to test if the model structure, together with prior information on the parameter values, can explain important features of the system being studied.

**Figure 2:**
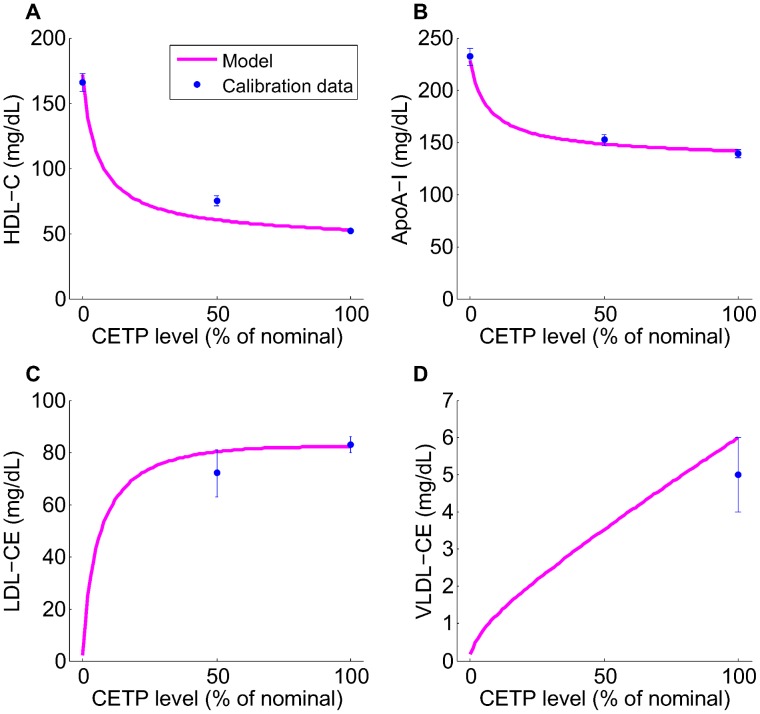
The fit of the model to the calibration data for CETP deficiency: HDL-C (panel A), ApoA-I (panel B), LDL-CE (panel C) and VLDL-CE (panel D). The data are as shown in Table 6, obtained by pooling HDL-C and ApoA-I data from references [81]–[83] and LDL-CE, VLDL-CE data from references [20], [21]. The model simulation curves were obtained by decreasing the 3 parameters representing CETP activity 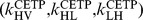 from 100% to 0% of those corresponding to the nominal subject.

**Figure 3:**
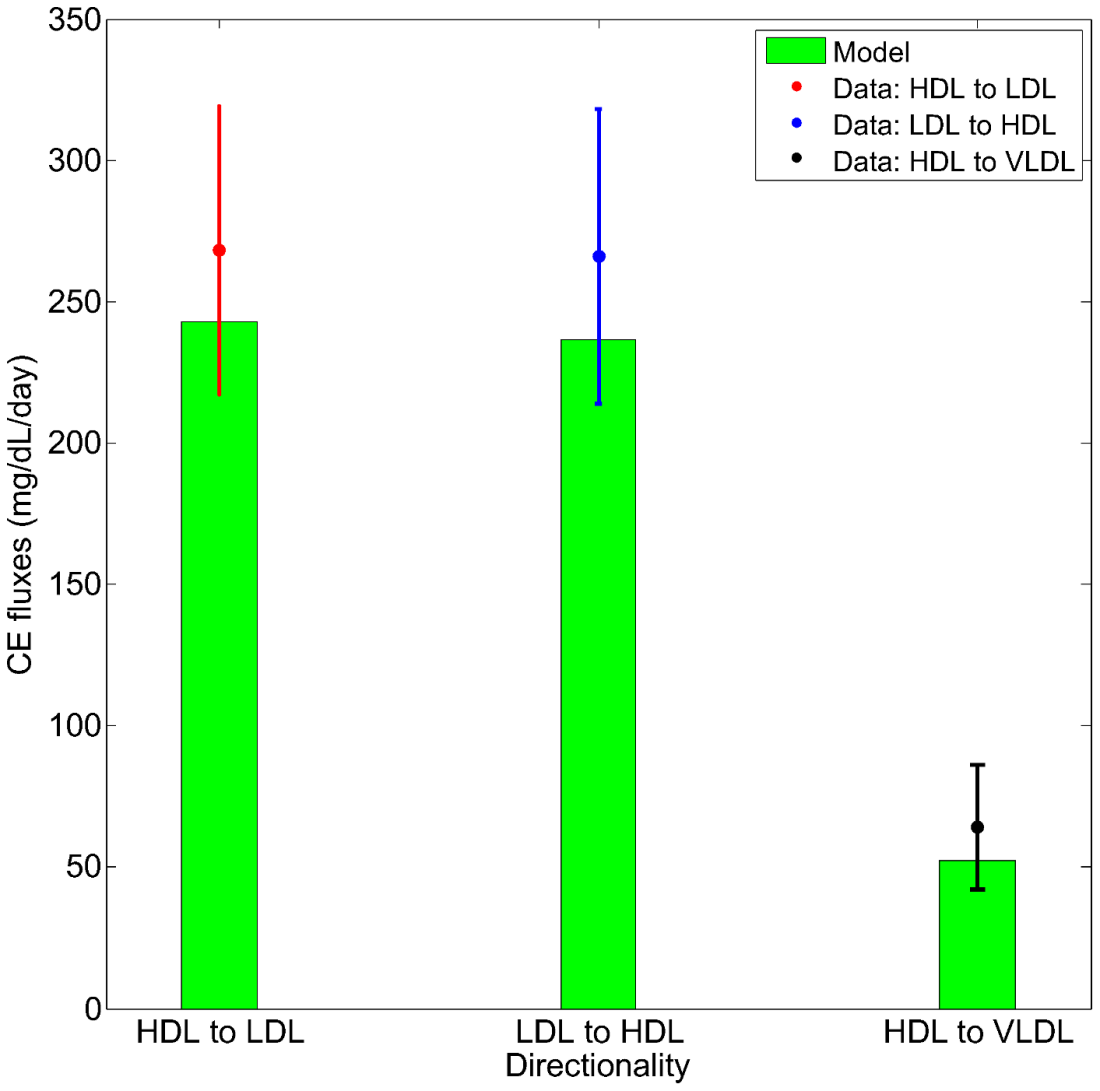
The fit of the model to the calibration data: CE fluxes. The data are as shown in Table 8, taken from reference [20]. The model simulation is produced using the point estimate of parameters for the nominal subject.

**Figure 4:**
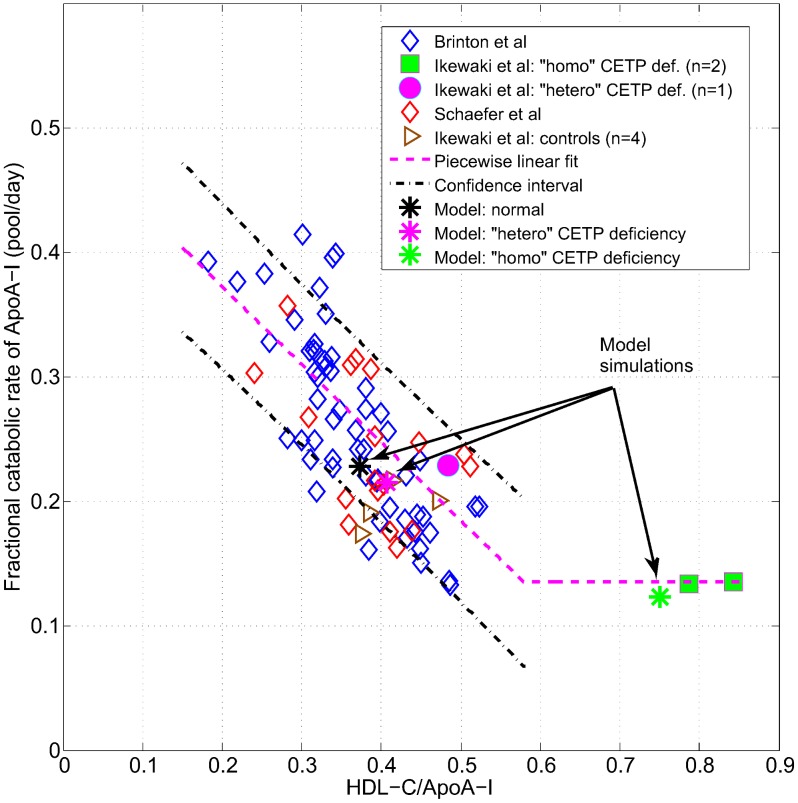
The fit of the model to the calibration data: FCR of ApoA-I versus HDL-C/ApoA-I ratio. The data sources are: Brinton *et al* [43], Ikewaki *et al* [45], Schaefer *et al* [44]. The piecewise linear fit and the confidence interval are discussed in the Methods section. The model simulation values are indicated by asterisk symbols, for the nominal subject and the heterozygote, homozygote of CETP mutation.

In our current work, the LMK model was used to explain the effects of CETP inhibition on ApoA-I level as well as the inverse relationship between the FCR of ApoA-I and particle size. Based on this, the calibration data were chosen. In Figures 2, 3 and 4 we show the calibration data superimposed with the model simulation, using the *maximum a posteriori* parameter set identified by the calibration procedure (as described in the Methods section). In Figure 2, the decrease in CETP level from 100% to 0% of the nominal subject was simulated by decreasing the three parameters associated with CETP activity 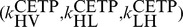 by the same factor. In particular, panels A and B show that the rise in HDL-C (the concentration of HDL-C is computed by summing 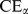 and free cholesterol pool, as discussed in the Methods section) and ApoA-I in heterozygotes and homozygotes with CETP deficiency are fairly well captured by the LMK model; the main discrepancy is the under-prediction of HDL-C for CETP heterozygotes. To our knowledge, the increase in ApoA-I under CETP deficiency or inhibition has not yet been explained by existing models: by incorporating the geometric ideas proposed in [25] the LMK model provides, for the first time, a way to connect the metabolism of ApoA-I and HDL-C. We remark that the LMK model is focused on HDL rather than the metabolism of ApoB-containing particles, which include LDL and VLDL. In particular, the LMK model predicts negligible concentrations of LDL-CE and VLDL-CE in CETP homozygotes, which are inconsistent with the reported concentrations in these subjects [39]–[41]. We believe that this discrepancy between the LMK model prediction and reality is due to *β*-LCAT activity [42], which in CETP homozygotes could compensate for the lack of CE influx from HDL particles by converting free cholesterol on the surface of ApoB-containing particles into cholesteryl ester. Finally, Figure 3 shows that the CE fluxes in the LMK model are consistent with the values measured [20], in particular the CE flux from HDL to LDL is close to that from LDL to HDL.

Another important and robust finding that has been observed in HDL metabolism is the relationship between FCR of ApoA-I and particle size (estimated using a surrogate measure) seen in normal subjects [43], [44]; in addition, heterozygotes and homozygotes of CETP deficiency are also observed to have a decreased FCR of ApoA-I [45]. Thus, an important objective of the calibration process is to test whether the structure of the model, together with an assumption on the linear size-dependence of HDL holo-particle uptake rate, can explain this relationship. The inverse relationship observed between the FCR of ApoA-I and the ratio HDL-C/ApoA-I (a surrogate measure of HDL size) is shown in Figure 4: in particular, data from Schaefer *et al* [44], Brinton *et al* [43] and Ikewaki *et al* [45] are given. A linear fit was carried out using the pooled data of Schaefer *et al* [44], Brinton *et al* [43] and normal subjects from Ikewaki *et al* [45], with the mean shown as a dashed pink line and the 1 SD confidence region shown as dashed black lines in Figure 4. The ApoA-I FCR for CETP homozygotes (who have large HDL particles) are assumed to be the lowest level attainable, hence this value was taken as the “floor” of the fit. The LMK model was calibrated to the piecewise linear relationship represented by the pink line and Figure 4 shows that simulations for normal and CETP mutation subjects (denoted by the asterisk symbols) are all in good agreement with the inverse relationship. The LMK model reproduces the dependence of ApoA-I FCR on CETP primarily by changing the distribution of ApoA-I between the lipid-poor and *α*-HDL pools (which have different clearance rates), with a minor contribution from the explicit size dependence of holo-particle uptake, 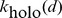.

### Model validation

**Figure 5:**
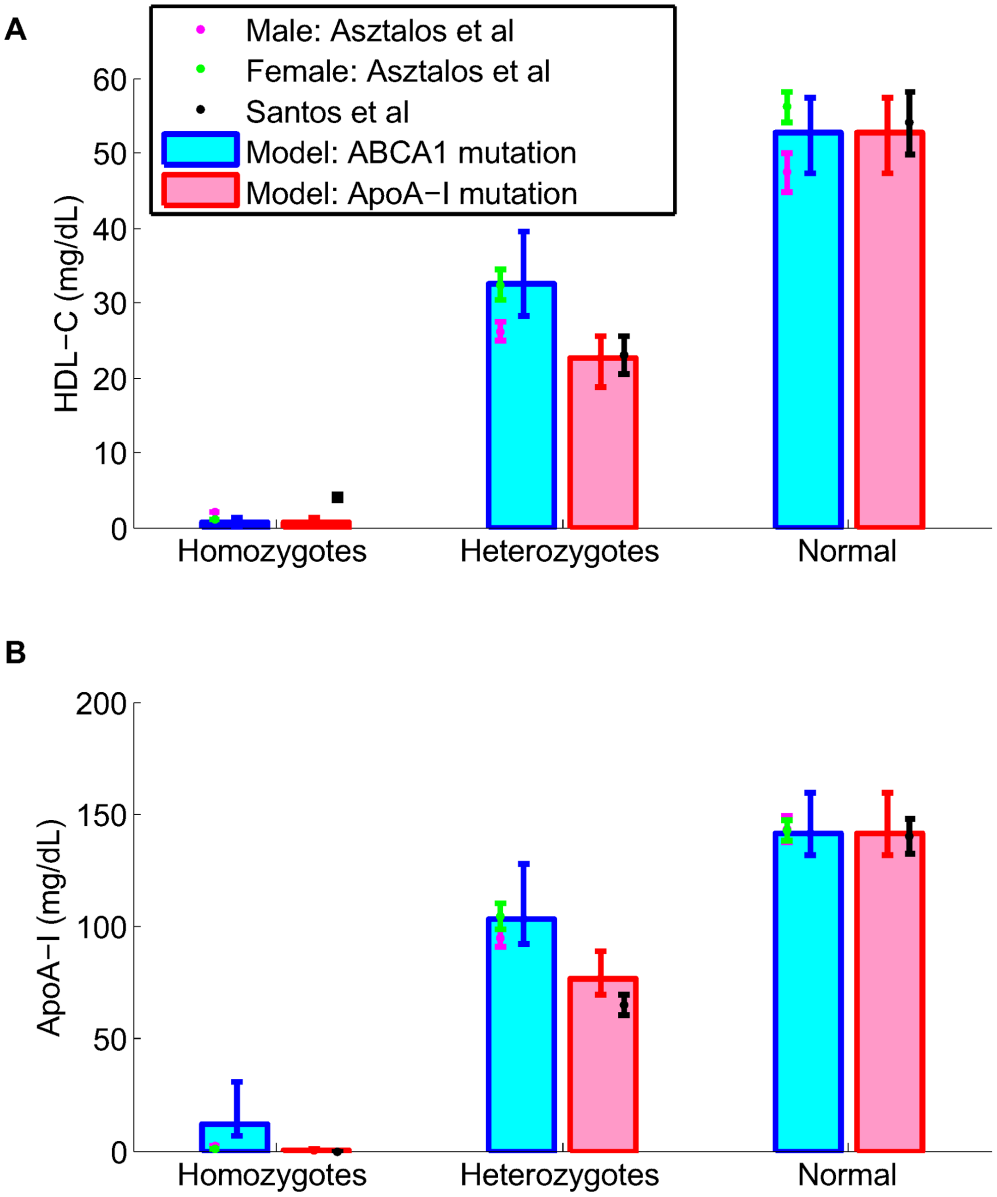
Model validation: simulation of ABCA1 and ApoA-I mutations compared with literature data for HDL-C (panel A) and ApoA-I (panel B). For the simulation results, the mean and the 95% confidence intervals are plotted. The data sources are Asztalos *et al* [46] and Santos *et al* [47]; the mean ± SD are shown. The model simulations of the mutation cases were obtained by taking the parameter values for the nominal subject and set 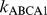 and 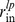 to 50% and 0% of the nominal values respectively.

In order to increase confidence in its predictions, the LMK model has been validated by simulating a number of scenarios that have not been used in the calibration process. In particular, since ABCA1 and ApoA-I are important targets in the pathway, the literature data on subjects with mutations in these genes [46], [47] are compared against the model simulations. The heterozygotes and homozygotes of ABCA1 mutation are simulated by setting 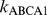 (representing ABCA1 activity) to 50% and 0% of its nominal value respectively; similarly, heterozygotes and homozygotes of ApoA-I mutation are simulated by setting the parameter 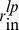 (representing ApoA-I synthesis rate) to 50% and 0% of its nominal value respectively. Figure 5 shows the mean and 95% confidence intervals of the model simulations, compared to the literature data (mean and SD are given). An examination of the results for the heterozygotes shows that, encouragingly, the LMK model is able to differentiate between the effects of ABCA1 and ApoA-I mutations on HDL-C and ApoA-I levels: both quantities decrease more for ApoA-I heterozygotes as compared to ABCA1 heterozygotes. Furthermore, the LMK model predicts that heterozygotes of ABCA1 mutation have smaller HDL particles (data not shown), consistent with the data of Asztalos *et al* [46].

**Figure 6:**
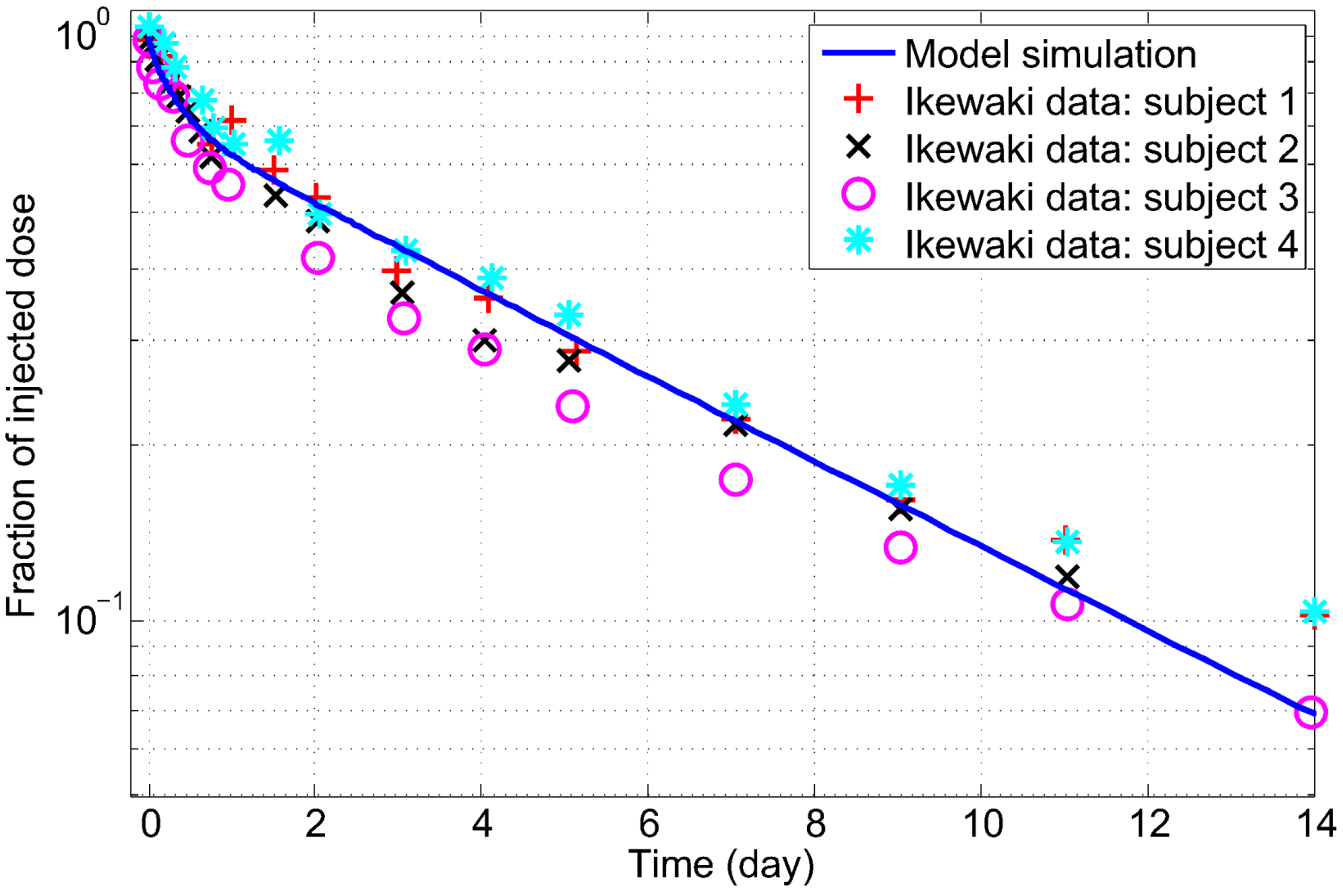
Model validation: Simulation of tracer kinetic experiment with labelled ApoA-I compared to experimental data. The data are obtained by digitization of tracer kinetics measurements carried out in 4 subjects and shown in Figure 3 of Ikewaki *et al* [48]. The model simulation corresponds to the nominal subject.

Most of the calibration data are static in nature, hence it is of particular interest to perform dynamic simulations of the LMK model and compare them to existing data. As a validation, we would like to see if the LMK model reproduces the characteristic biphasic decay curves seen in tracer kinetic experiments with labelled ApoA-I. In the LMK model, the injection of radio-labelled dose is represented by a small addition to the pool of lipid-poor ApoA-I and the fractional dose remaining in the sum of the two pools of ApoA-I is plotted; refer to the Methods section for the details of the simulation methodology. This is simulated using the parameters identified for the nominal subject and the result is shown in Figure 6: it can be seen that the simulated decay curve is biphasic and similar to the data obtained by digitizing Figure 3 of Ikewaki *et al* [48]. Furthermore, the mean residence time (which is the inverse of FCR) of labelled ApoA-I computed from the model simulation is 4.2 days, which is in good agreement with the result of 4.8±0.3 days as measured in 4 subjects by Ikewaki *et al* [48].

### Explaining epidemiological relationship using a virtual population

Having calibrated and validated the LMK model, we use it as a platform for exploring the observed epidemiological relationship between HDL-C and CVD risk. For this purpose, a virtual population is generated in a manner analogous to that of reference [49]. In particular, model parameters are sampled from a multivariate normal distribution and for each set of parameters the “phenotype” of the corresponding virtual subject is simulated using the LMK model. As there is no information available on the correlation between model parameters in a real population, we have assumed them to be uncorrelated and each is drawn from a normal distribution with a relative SD = 15% around the value corresponding to the posterior values for the nominal subject (see Table 5).

**Figure 7:**
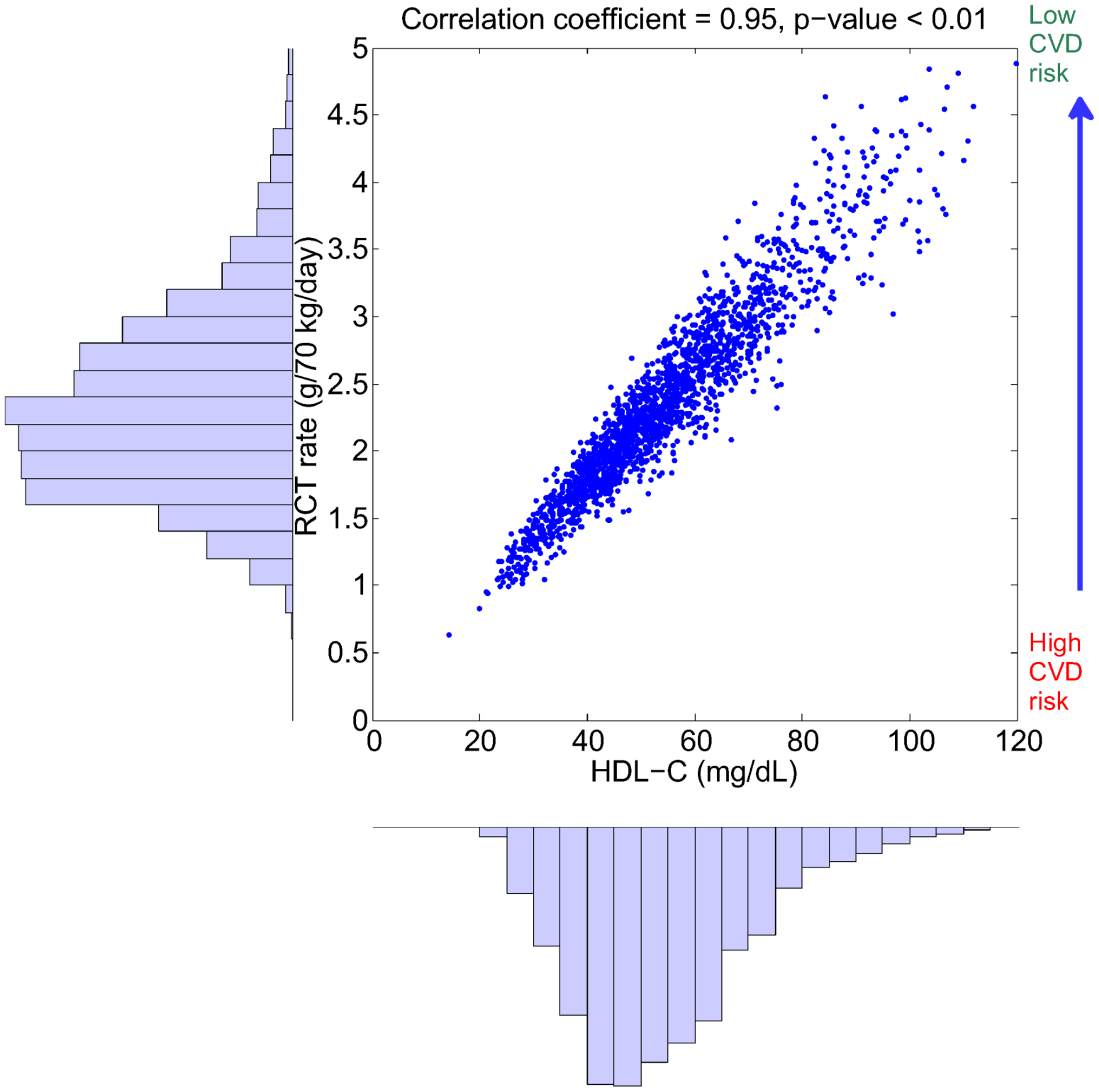
The distribution of RCT rate and HDL-C and their correlation in the simulated virtual population. By drawing the parameters of the model from an uncorrelated, multivariate normal distribution, a set of 2000 virtual patients is generated and the model simulations of RCT rate and HDL-C are shown. The right-hand axis represents the hypothetical inverse relationship between RCT rate and CVD risk.

**Figure 8:**
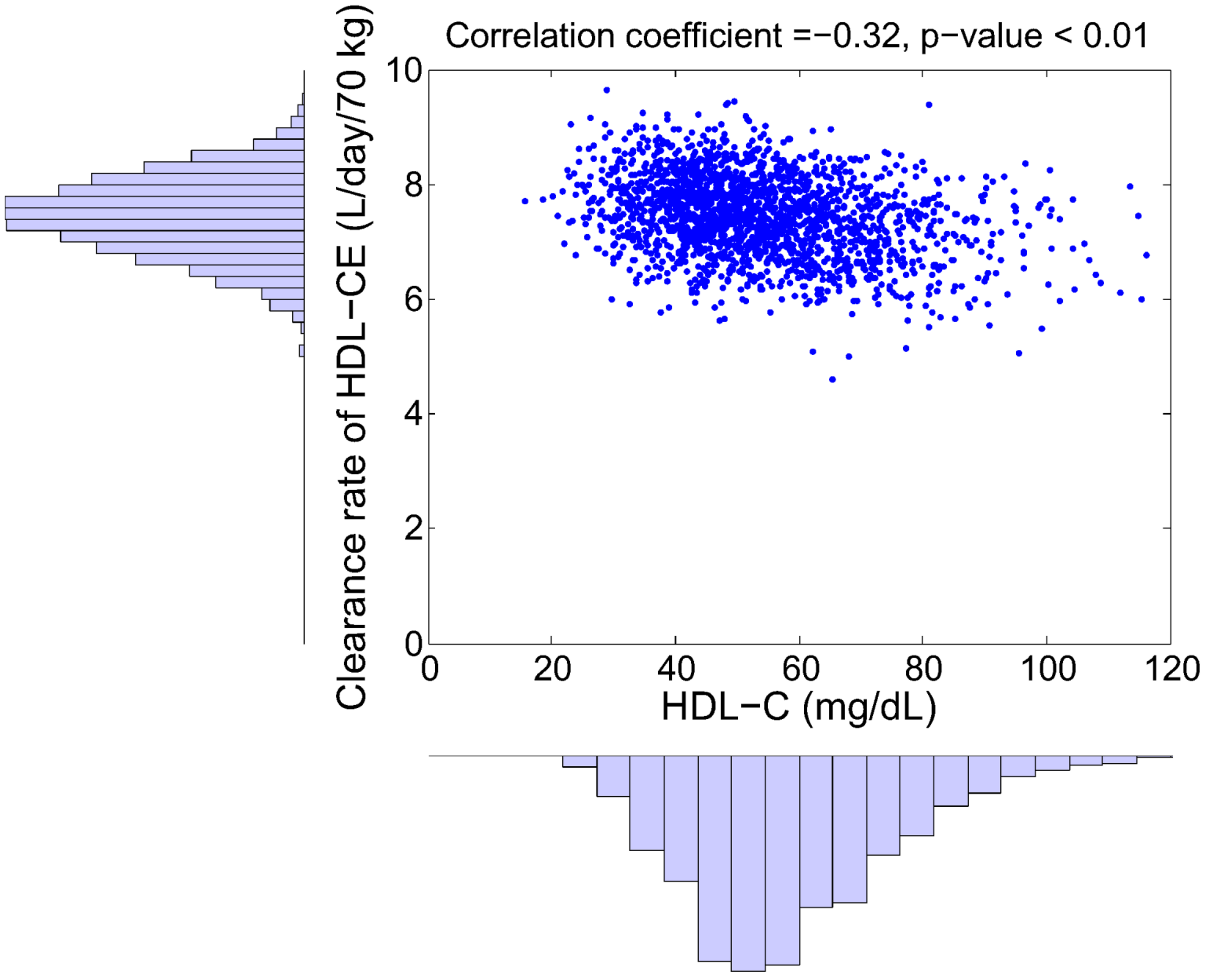
The distribution of the clearance of HDL-CE and HDL-C and their correlation in the simulated virtual population. By drawing the parameters of the model from an uncorrelated, multivariate normal distribution, a set of 2000 virtual patients is generated and the model simulations of HDL-CE clearance rate and HDL-C are shown.

Despite the fact that the parameter distribution in the virtual population is uncorrelated, some of the simulation outputs show significant correlations as a result of the model structure. Of particular interest is the correlation between RCT rate (as defined in (2)) and plasma biomarkers. Shown in Figure 7 is the relationship between RCT rate and HDL-C within the virtual population: it can be seen that there is a surprisingly strong correlation between the two quantities (*r* = 0.95). We note that the RCT rate given in (2) corresponds to the input rate of HDL-CE into plasma: in fact, the plasma concentration of HDL-CE can be expressed as the following:
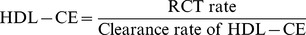
(3)where the clearance is defined as the plasma volume multiplied by the sum of elimination rate constants. In the LMK model, elimination processes for HDL-CE include those mediated by CETP and SRB1, as well as the holo-particle uptake. While the RCT rate shows a strong correlation with HDL-C, we see that in Figure 8 the clearance of HDL-CE shows a much weaker negative correlation with HDL-C (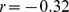). Hence, the simulation results suggest that the variation in HDL-C within the virtual population is largely attributed to variations in the RCT rate and not due to its clearance. Under the “HDL flux hypothesis” [2] that low RCT rate results in high CVD risk, the relationship shown in Figure 7 provides a plausible explanation for the epidemiological association between HDL-C and CVD risk. The same set of virtual subjects is also used in subsequent sections for target evaluation and biomarker identification.

### HDL-C raising therapies

The LMK model can be used to evaluate actual and potential HDL-C raising therapies, by modulating targets of interest. We have used simulated the model for both the nominal subject, as well as for a virtual population.

#### CETP inhibition

**Figure 9:**
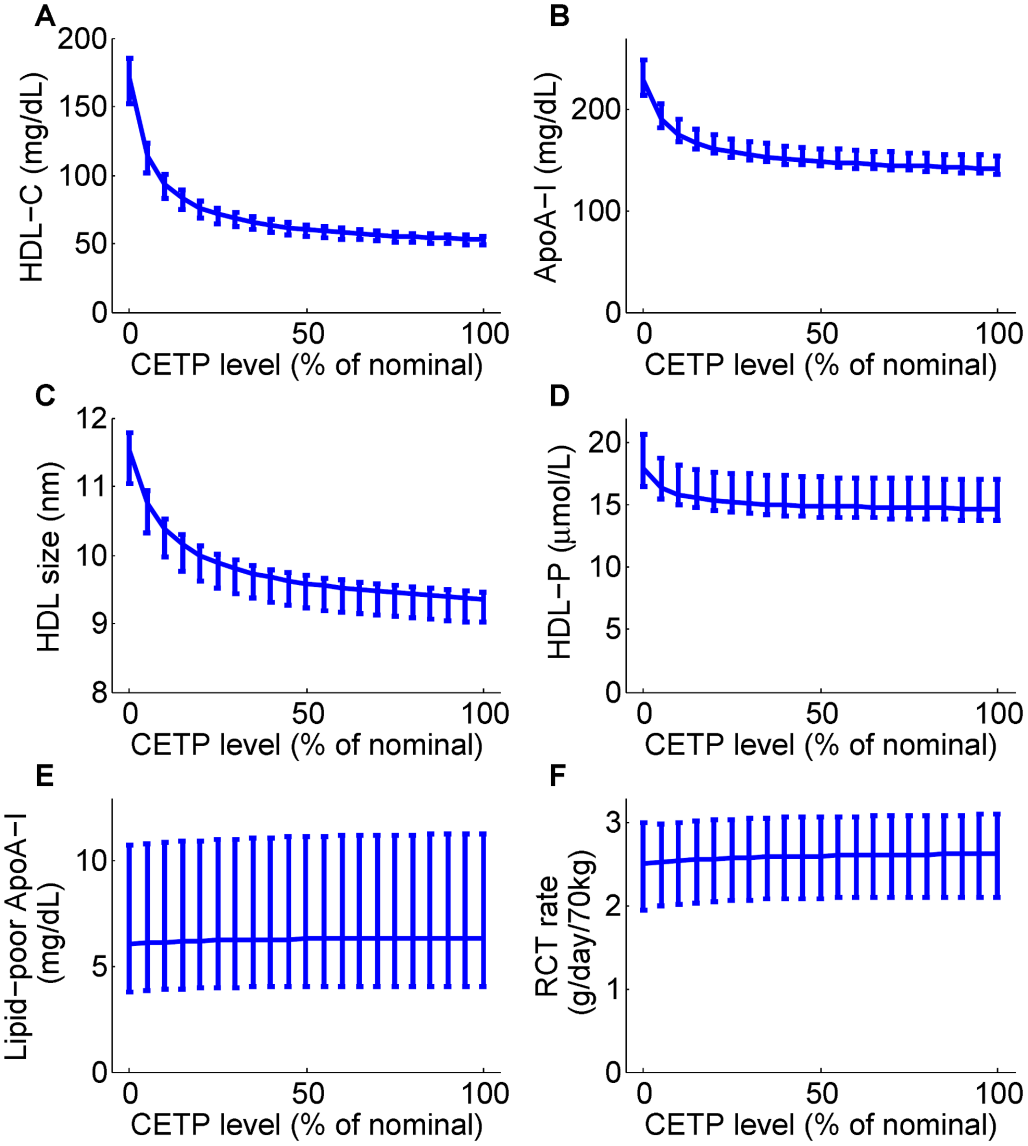
Model predictions for the dependence of HDL measures (HDL-C, panel A; ApoA-I, panel B; HDL size, panel C; HDL particle concentration, panel D; lipid-poor ApoA-I, panel E) and RCT (panel F) on the CETP level. The model simulation curves were obtained by decreasing the 3 parameters associated with CETP activity 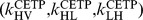 from 100% to 0% of those corresponding to the nominal subject. For each prediction, the mean and the 95% confidence intervals are plotted.

The model predictions of CETP inhibition on the nominal subject, together with 95% confidence intervals, are shown in Figure 9. In alignment with the calibration data, as CETP level decreases both HDL-C and ApoA-I increase strikingly (see panels A and B). These changes are associated with a significant increase in HDL size as well as a small increase in HDL particle concentration (HDL-P) (see panels C and D). Both the absolute concentration of lipid-poor ApoA-I and RCT rate remain essentially unchanged (see panels E and F).

**Figure 10:**
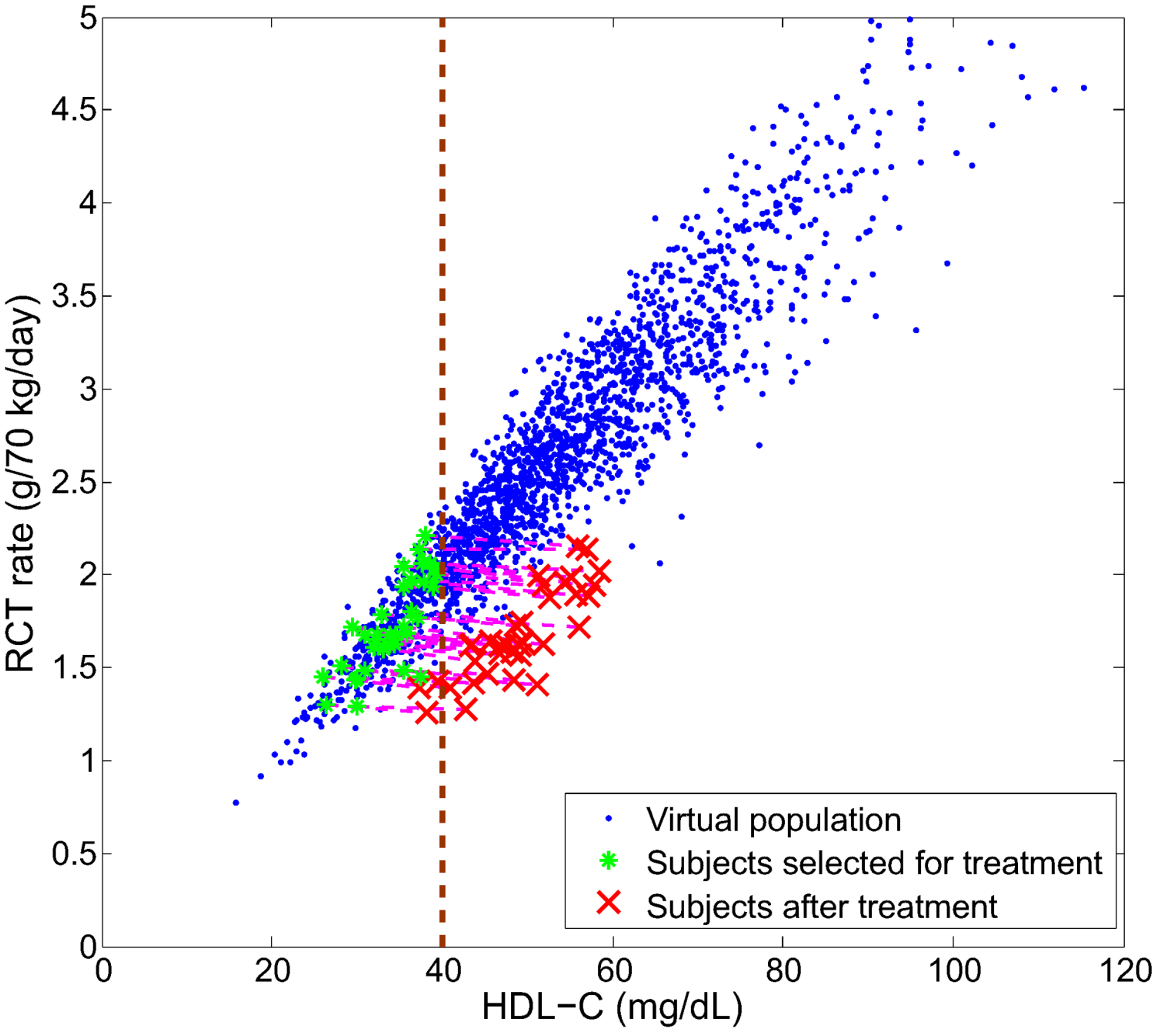
Simulation of CETP inhibition on a virtual population with low HDL-C (≤40 mg/dL). Each virtual patient selected for the treatment simulation had its rate constants associated with CETP activity 
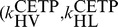
 and 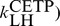 decreased to 20% of their original values.

To further illustrate the effect of CETP inhibition, in Figure 10 we simulate the therapy in a virtual population. In particular, we select subjects with low HDL-C (40 mg/dL or less) for treatment with a hypothetical drug that inhibits the plasma CETP by 80% and simulate the changes in HDL-C and RCT rate in the treated subjects. It can be seen that the rise in HDL-C does not correspond to an increase in RCT rate. In fact, the effects induced by CETP inhibition depart from the baseline relationship. This is an illustration of a target impacting a biomarker which is correlated but not causally linked with the disease mechanism: the hypothetical drug does not bring about a therapeutic effect of increasing RCT rate despite increasing HDL-C. However, there could be a potential CV benefit due to a small (14%) decrease in LDL-C (data not shown).

#### ABCA1 up-regulation

**Figure 11:**
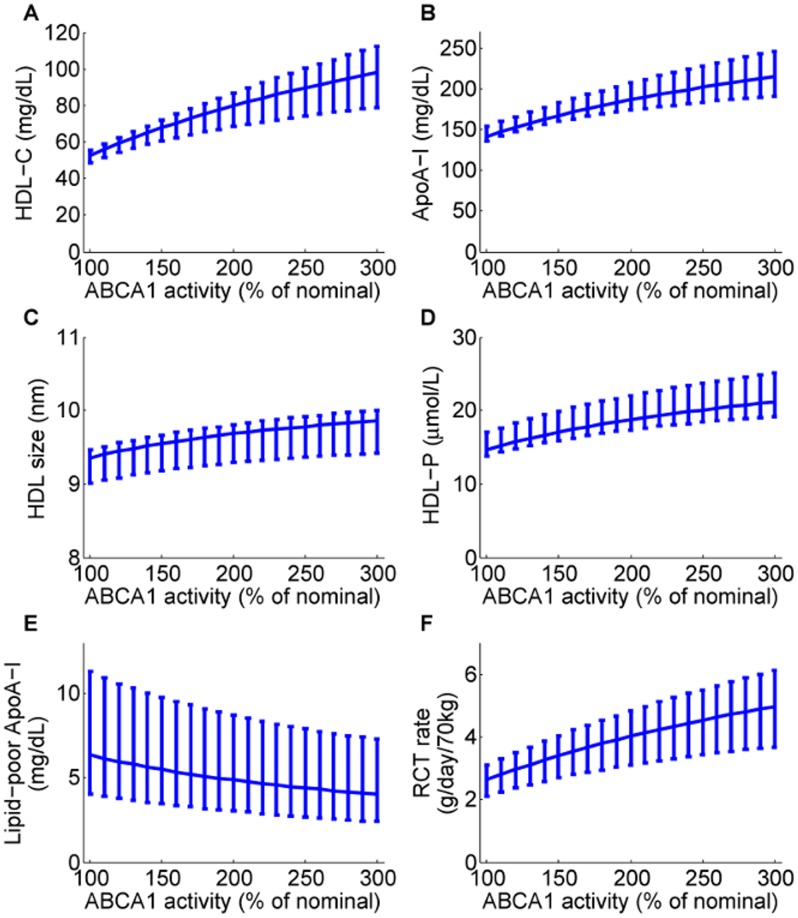
Model predictions for the dependence of HDL measures (HDL-C, panel A; ApoA-I, panel B; HDL size, panel C; HDL particle concentration, panel D; lipid-poor ApoA-I, panel E) and RCT (panel F) on ABCA1 activity. The model simulation curves were obtained by increasing the parameter representing ABCA1 activity 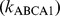 from 100% to 300% of the nominal subject. For each prediction, the mean and the 95% confidence intervals are plotted.

The model predictions for ABCA1 up-regulation on the nominal subject, together with 95% confidence intervals, are shown in Figure 11. The simulation results show that as ABCA1 activity increases, both HDL-C and ApoA-I increases (see panels A and B). Panels C and D of Figure 11 show that these increases reflect not only an increase in HDL size, but also increases in particle concentration. In stark contrast to CETP inhibition, under ABCA1 upregulation the RCT rate is predicted to increase markedly (panel E) and the absolute concentration of lipid-poor ApoA-I decreases.

**Figure 12:**
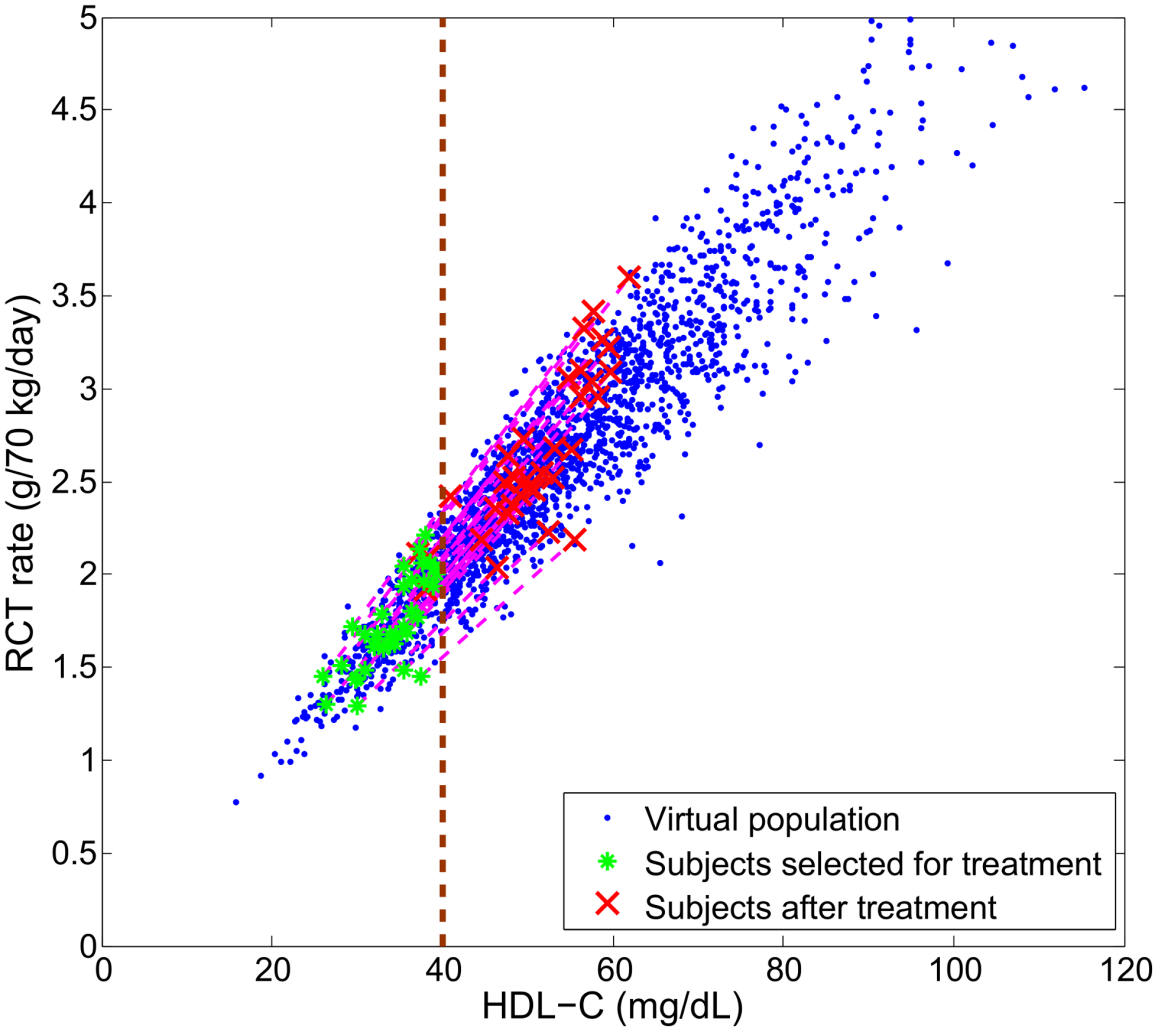
Simulation of ABCA1 up-regulation on a virtual population with low HDL-C (≤40 mg/dL). Each virtual patient seleted for the treatment simulation had its ABCA1 activity (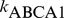) increased by 100% of its initial value.

We next consider ABCA1 up-regulation for the virtual population as shown in Figure 7. In particular, we select the same subjects with low HDL-C as was previously chosen for CETP inhibition. We simulate a hypothetical drug that increases ABCA1 activity in each of the treated subjects by 100% and examine the changes in HDL-C and RCT rate. As shown in Figure 12, under ABCA1 up-regulation both HDL-C and RCT rate increase. In particular, the changes induced by ABCA1 up-regulation are predicted to follow the baseline epidemiological relationship.

**Figure 13:**
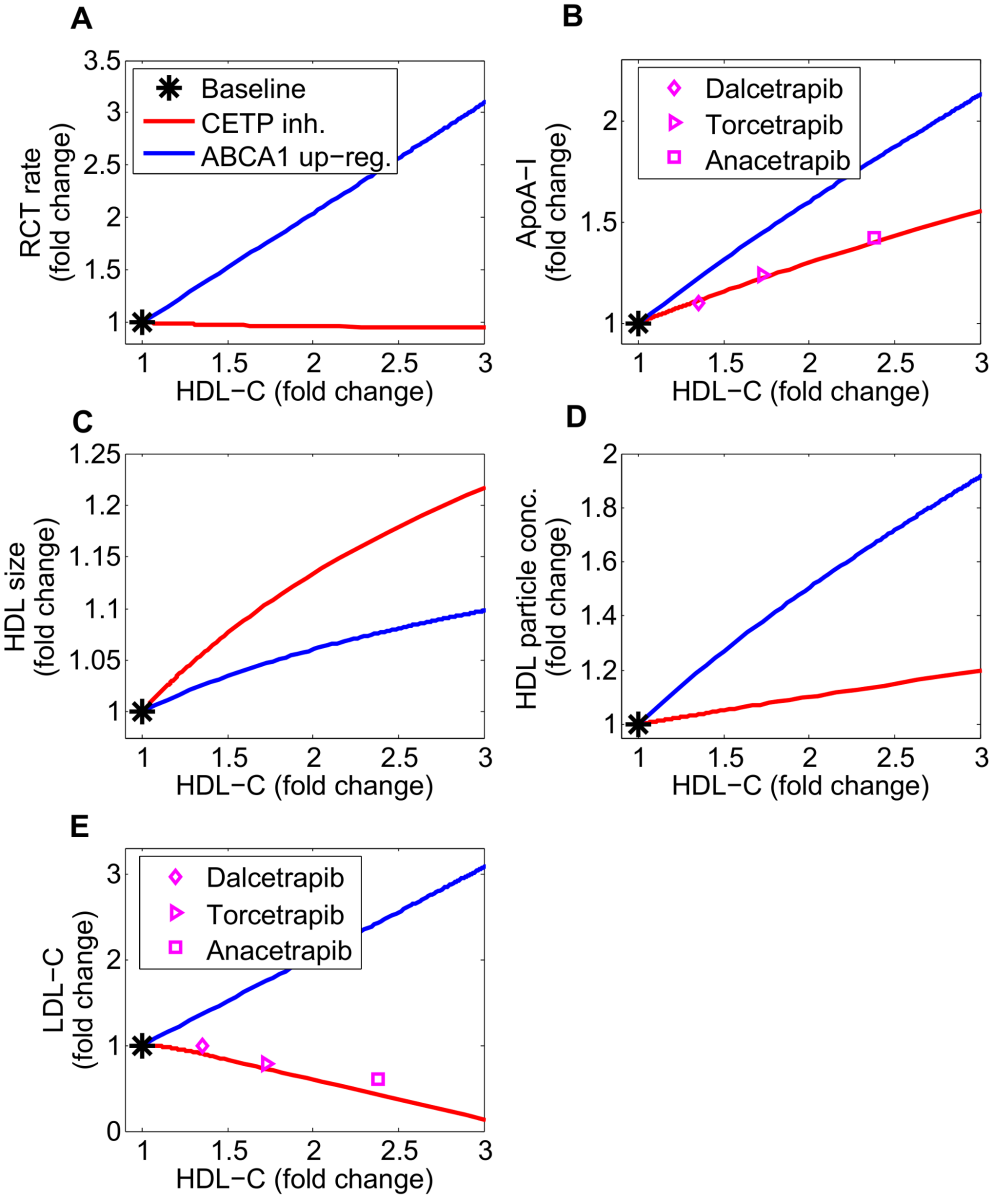
Comparison of CETP inhibition with ABCA1 up-regulation: changes in RCT rate (panel A) and biomarkers (ApoA-I, panel B; HDL size, panel C; HDL particle concentration, panel D; LDL-C, panel E) versus the rise in HDL-C. The nominal subject is taken as the baseline. The model simulation of CETP inhibition is compared with literature data of CETP inhibitors, Dalcetrapib [50], Torcetrapib [51] and Anacetrapib [52].

In order to further elaborate on the differences between the two target modulations, we compare in Figure 13 the changes in biomarkers for CETP inhibition and ABCA1 up-regulation. The simulation results show that for a given fold change in HDL-C, CETP inhibition gives rise to larger particle sizes but fewer particle numbers as compared to ABCA1 up-regulation. Due to the differences in the particle size and number under the two target modulations, for a given fold-change in HDL-C, ApoA-I is predicted to increase more under ABCA1 up-regulation as compared to CETP inhibition. Reassuringly, the simulated increases in ApoA-I for CETP inhibition are in fair agreement with literature data for the three CETP inhibitors, Dalcetrapib [50], Torcetrapib [51] and Anacetrapib [52]. For CETP inhibition the predicted decline in LDL-C as the fold-change in HDL-C increases is in good agreement with data on the three CETP inhibitors (Figure 13, panel E). Conversely for ABCA1 up-regulation the LMK model predicts an increase in LDL-C with increasing fold-change in HDL-C. This finding is a consequence of the first-order CETP-mediated transfer processes between HDL, VLDL and LDL particles. It is qualitatively consistent with the GWAS study of Voight *et al* [53] which showed that ABCA1 SNP rs3890182 raised HDL-C and LDL-C by comparable amounts.

### Lipoprotein biomarkers

A number of studies have shown that CVD risk is correlated with plasma biomarkers such as HDL-C [1], HDL-P [54] and pre-*β*_1_ [55], [56] levels. In addition, the combination of NMR analysis of HDL with genotyping has also given a glimpse into the possible genes associated with HDL particle measures [57]. However, the mechanistic basis for these experimental observations as well as what underlies the correlations between the plasma biomarkers are not well understood. Using the proposed LMK model, we can reproduce and explain the correlations between these plasma biomarkers.

**Figure 14:**
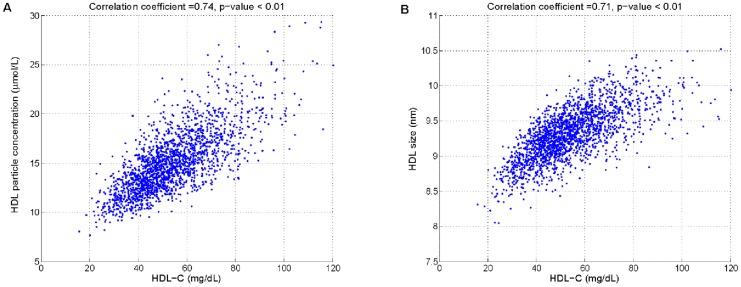
Correlations between HDL-C and HDL-P (panel A), and HDL-C and HDL size (panel B) in a virtual population of 2000 subjects.

In addressing these questions, the simulated biomarkers within the population of 2000 virtual patients (as previously shown in Figure 7) were studied. The correlation between HDL-P and HDL-C within this set of virtual patients (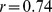) is shown in Figure 14, panel A; we see that the simulation result is qualitatively similar to the positive correlation shown by Mackey *et al* [54] (the absolute values of HDL-P obtained by NMR are approximately 2-fold greater than our simulations which are based on the updated Shen model; the discrepancy is discussed in [25]). A positive correlation also exists in the virtual population between HDL size and HDL-C (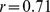), consistent with Mackey *et al* [54] (see Figure 14, panel B).

Due to the growing appreciation for the importance of RCT [2], [8], [58], there are on-going efforts in trying to quantitatively assess the steps involved in the process. The ABCA1 transporter is involved in the first step of RCT by removing cholesterol from peripheral tissues to plasma and its activity level in patients has been studied [59]. In particular, ABCA1 gene expression and protein concentration on leukocytes has been measured in patients with type 2 diabetes, where the data suggested a negative correlation between ABCA1 expression and HbA1c levels [59]. While there are assays that can quantify ABCA1 protein levels in specific cell types [60], an experimental technique for the assessment of ABCA1 activity *in-vivo* at the whole body level has yet to be developed. Given the current experimental limitations, there is an interest to evaluate the potential effectiveness of plasma-based biomarkers for quantitatively assessing ABCA1 activity.

**Figure 15:**
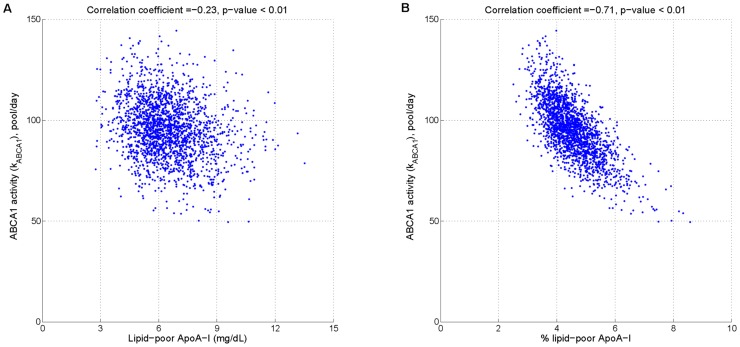
Correlations between 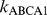 and absolute concentration of lipid-poor ApoA-I (panel A), and between 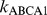 and % lipid-poor ApoA-I (panel B) in a virtual population of 2000 subjects.

**Figure 16:**
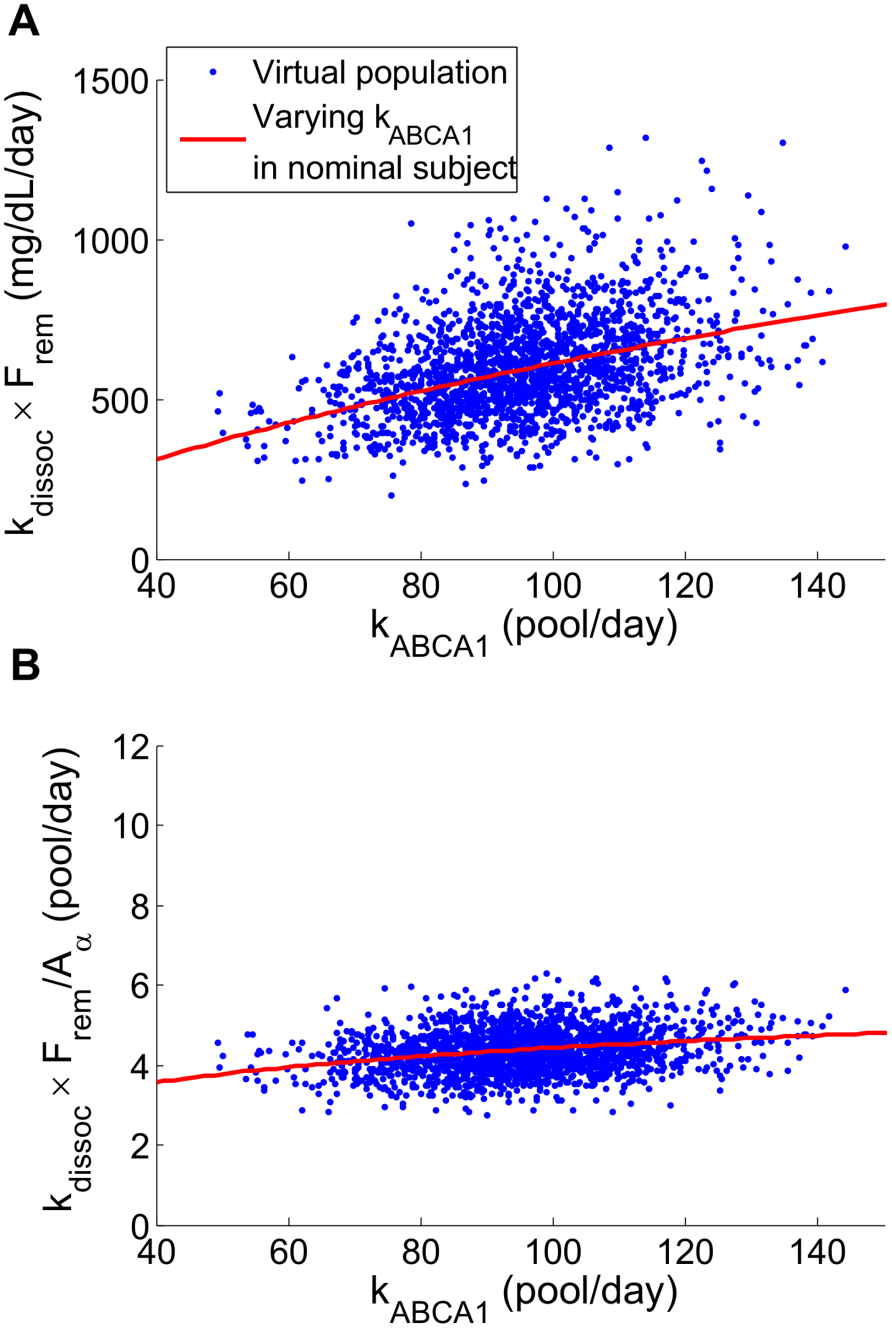
Comparison of two expressions involving remodeling flux: 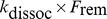 (panel A) and 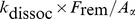 (panel B). The distributions of absolute and ApoA-I adjusted remodeling flux in the virtual population are plotted against 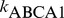. The simulations of the nominal subject with only the parameter 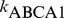 varied are shown as solid lines.

Using the LMK model, we evaluated the potential effectiveness of two biomarkers for ABCA1 activity: firstly, the absolute concentration of lipid-poor ApoA-I; secondly, the relative concentration of lipid-poor ApoA-I as the percentage of total ApoA-I. Figure 15 panel A shows that the former is only weakly correlated with ABCA1 activity. In contrast, panel B shows that the latter exhibits a strong inverse correlation with ABCA1 activity; in fact, given a measured value of % lipid-poor ApoA-I, the relationship can be used to estimate ABCA1 activity. This result can be better understood by the following analysis. From equation (1a), the absolute concentration of lipid-poor ApoA-I at steady state can be expressed as:
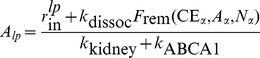
(4a)

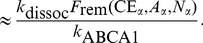
(4b)On the other hand, from (1b) the % lipid-poor ApoA-I can be expressed as the following:
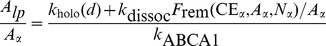
(5a)

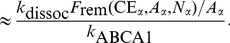
(5b)Comparison of the denominators in (4a) and (5a) show that in the former expression, an additional parameter 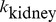 enters; however, it is small compared to 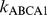 (the mean values being 2.42 and 95.18 respectively; see Table 5). In the numerator, the main quantitative difference between the two expressions is the remodeling flux, 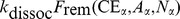, versus the ApoA-I normalized flux, 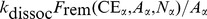. As shown in Figure 16, the latter has a flatter dependence on 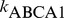 as well as less variability due to other parameters. As a result, the ratio 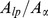 allows for a more precise estimate of 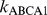 compared to 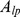 above. In conclusion, the analysis shows that the stronger inverse relationship shown in Figure 15 panel B can be attributed to the normalization of the remodeling flux by ApoA-I.

The simulation results may further explain why, in some literature studies, % lipid-poor ApoA-I (note that the absolute concentration of lipid-poor ApoA-I can be experimentally estimated by assays that measure pre-*β*_1_ [61]) has been proposed as a risk factor, as well as how increased % lipid-poor ApoA-I could be associated with CVD risk. Our proposal of using the % lipid-poor ApoA-I as a surrogate measure for ABCA1 activity is in concordance with the previous suggestion by Asztalos *et al* [62] that the ratio pre-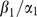 is a measure of the efficiency of RCT: a decrease in this ratio has been thought to reflect an enhanced RCT [62], [63]. In addition, our finding of the inverse correlation between % lipid-poor ApoA-I and ABCA1 activity may explain the observation that increased fractional pre-*β*_1_ is associated with increased maximum intima-media thickness in both diabetics [64] and non-diabetic subjects [65], as well as being associated with an increased risk for coronary heart disease and myocardial infarctions [66].

### Future directions

We foresee a number of potential future applications of the LMK model in the context of drug discovery and development, including the following:

1. Confirmation of a molecule's mechanism of action: this can be done by checking the clinically observed changes in lipoprotein measures against the model predictions. This is an important task, since a molecule that increases HDL-C may do so by modulating the RCT pathway not only on its intended target but may also have off-target effects. As the model shows, the choice of mechanism in raising HDL-C could be crucially important for whether or not it brings about cardiovascular benefit.
2. Determining the right dosage schedule for maximum cholesterol removal: the LMK model could help to integrate the pharmacokinetics of a molecule with the dynamics of HDL metabolism.
3. Evaluating combinations of target modulations: the LMK model could help to address the question of the potential synergism between targets in the RCT pathway.
4. Development of personalized health care (PHC) strategy: simulations of the model to generate virtual populations could be used to address the question of which patient subpopulations are most likely to benefit from a given therapy and how those subjects might be selected using plasma-based diagonostic tests.

The LMK model is focused on capturing the dynamics of ApoA-I and CE transfers. However, extensions of the model to incorporate ApoA-II dynamics as well as explicitly representing triglyceride and phospholipid metabolism would be important for describing the effects of other drug classes, including the PPAR-*α* and *γ* agonists [67], [68] or synthetic phospholipids [69], [70]. These remain topics for further research.

### Conclusions

We have developed a novel, *in-silico* model of lipoprotein metabolism focused on the reverse cholesterol transport pathway. The model incorporates important concepts of HDL biology, including the regeneration of lipid-poor ApoA-I via *α*-HDL remodeling processes, and has been calibrated using literature data from a wide variety of sources. The model has been further validated by simulating scenarios not considered in the calibration process. These include its ability to reproduce the levels of HDL-C and ApoA-I in hetero- and homozygous subjects with either ABCA1 or ApoA-I mutation and the observed biphasic kinetics of ApoA-I seen in tracer kinetics studies. This provides an increased confidence in the LMK model predictions with respect to modulations of these important targets and in the model's ability to simulate time-dependent scenarios.

In this paper, we have illustrated the applications of the LMK model in comparing the two target modulations, CETP inhibition and ABCA1 up-regulation. The results drawn from our model provide a possible explanation for the non-efficacy of dalcetrapib in the dal-OUTCOMES trial [7] as well as suggesting that ABCA1 is a target that would increase the RCT rate. The model provides predictions on the biomarker changes as a result of ABCA1 target modulation. Furthermore, computational experiments using a virtual population have shown why the % lipid-poor ApoA-I, rather than the absolute concentration of lipid-poor ApoA-I, is a better biomarker for assessing the *in-vivo* ABCA1 activity. By integrating mechanistic concepts and data, the model provides a way to quantitatively evaluate and explore hypotheses of lipoprotein metabolism.

## Methods

### Model derivation

#### Mass balance considerations

The LMK model (Figure 1) explicitly represents the mass balance of ApoA-I and CE molecules in plasma, whereas the mass balance of FC and PL molecules is represented implicitly. The input of ApoA-I to plasma reflects its synthesis rate, while the elimination of ApoA-I results from the excretion of lipid-poor ApoA-I by the kidney and holo-uptake of *α*-HDL particles by the liver. The remodeling of HDL particles by particle fusion, CETP, SRB1 and other processes leads to the recycling of ApoA-I from *α*-HDL particles to lipid-poor ApoA-I. Recycling influences the kinetics of ApoA-I in plasma but does not affect its mass balance. The input of CE to plasma reflects the rapid esterification of FC molecules in the nascent discs as they are converted to nascent spheres plus a small amount of CE which enters plasma during VLDL synthesis. The rate at which CE molecules appear in the *α*-HDL pool (via the nascent sphere) is defined in the LMK model as the RCT input rate and is assumed to equal the rate at which FC molecules are loaded onto the nascent discs (equation (2)). Elimination of CE from plasma results from holo-uptake and SRB1-mediated uptake of CE from all lipoprotein species. The CETP-mediated transfer of CE between *α*-HDL, VLDL and LDL does not affect the overall mass balance in plasma.

FC and PL molecules are present on the surfaces of all spherical lipoprotein particles in plasma as well as on the membranes of red blood cells (RBCs) and other cells that are in contact with the plasma. Based on Shen€s model of lipoprotein structure (Shen *et al* [71] and Mazer *et al* [25]), we assume that FC is in rapid equilibrium between all of these species and that the amount of FC present on each particle surface (at equilibrium) is dependent only on the surface curvature, that is, the radius of the hydrophobic core of the particle (as represented in equation (10), below). The FC needed for the surface of the nascent spheres is assumed to be provided by the large pool of FC present in blood, including RBCs, and is largely replenished by the HDL remodeling processes. It can be shown that the rate at which FC is eliminated from plasma via holo-uptake of HDL particles is very small compared to the RCT rate (<4%) and is therefore negligible from the perspective of mass balance. Similar considerations apply to the mass balance of PL.

#### Particle size

The size of spherical *α*-HDL particles is computed in the model as follows. From the pool size of CE in *α*-HDL and the particle concentration, the number of CE molecules per HDL particle is given by:
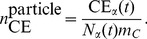
(6)Since 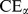 is expressed as an equivalent mass of FC and 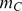 is the molecular weight of FC, 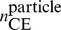 is appropriately determined. With an assumed ratio of TG/CE = 0.13 in the core of HDL particles [25], we sum the volumes occupied by CE and TG to obtain the total core volume and determine the core radius (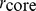) from it. Finally, the surface thickness 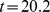 Å is added to the core radius, giving rise to following expression for the particle diameter, *d* (in Å):
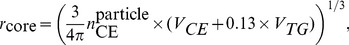
(7)

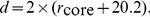
(8)Note that the molecular volumes 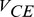 and 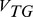 are defined in Table 3.

#### Remodeling flux

In the derivation of the remodeling flux, we compute the excess (or deficit) of ApoA-I compared to that derived using the updated Shen's model [25]. Given the pool size for 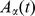 and the particle concentration 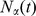, we compute the number of ApoA-I molecules per particle:
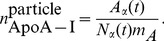
(9)Using the expression for 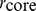 given in (7), the number of ApoA-I molecules needed to cover the surface is derived in the following manner [25]: firstly, the number of free cholesterol is computed,
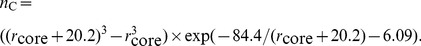
(10)Then, the number of phospholipid molecules needed to cover the remaining surface area of the core is computed:
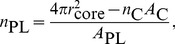
(11)where the cross-sectional surface areas of cholesterol and phospholipid are 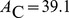 Å^2^ and 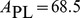 Å^2^ respectively. The number of amino acids needed to cover the hydrophobic area exposed at the outer surface layer of HDL particle is:
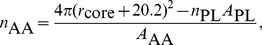
(12)where the cross-sectional area of an amino acid 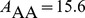 Å^2^. Since there are 243 amino acids in ApoA-I, under the further assumption that the weight fraction of ApoA-I in the HDL proteome is 60% [25], the number of ApoA-I molecules needed to cover the surface of HDL is given by:
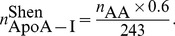
(13)Finally, the discrepancy between the number of ApoA-I on the HDL (9) and the number needed from the Shen model (13), is the excess (or deficit) ApoA-I. Given the HDL particle concentration 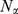, the following is the concentration of ApoA-I on *α*-HDL which is available to dissociate as the remodeling flux:
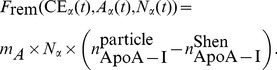
(14)


#### HDL holo-particle uptake

Our model allows for the possibility of a linear size dependence of the HDL holo-particle uptake rate. However, no prior assumption is made regarding the size dependency; using the calibration data, the sign and magnitude of the linear dependence is determined. In particular, the rate of holo-uptake has the following form, where the calculation of size *d* is given by equation (8) and the division by 10 accounts for the conversion from Å to nm:
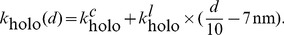
(15)


### Parameter priors

In this section, prior estimates of model parameters are given, including references to the original literature and the rationale for the choice of prior and the level of uncertainty. In a manner similar to a previously proposed Bayesian approach [35], uncertainty is increased by a factor 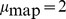 in the following cases:

- quantities that are measured *in-vitro* and mapped to the *in-vivo* context;
- quantities that are measured in a population with mutation(s) and mapped to normal subjects;
- quantities that result from pooling data obtained using distinct experimental techniques/assumptions.

No explicit prior correlations are assumed.

#### Synthesis rate of ApoA-I (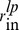)

The kinetics of ApoA-I were measured in *n* = 20 (11 males, 9 females) healthy subjects, with mean HDL-C = 46 mg/dL and ApoA-I = 115 mg/dL [44]. The ApoA-I synthesis rate has been estimated to be 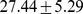 mg/dL/day (mean±SD). Hence, we take 
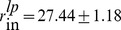
 mg/dL/day (mean±SEM).

#### Rate of kidney elimination (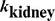)

There have been a number of papers describing the measurement of the FCR of pre-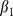 [22], [72]. However, the quantification of pre-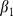 can be a challenging task and a more direct assessment of the clearance rate of lipid-poor ApoA-I is estimated by the FCR of ApoA-I in Tangier patients. In the model representation of homozygous Tangier patients (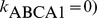, the FCR of ApoA-I equals the kidney clearance of lipid-poor ApoA-I. The hypothesis that kidneys is responsible for a large fraction of ApoA-I clearance is supported by the study of Braschi *et al* done using rabbits [73], where it was estimated that the kidneys contribute around 70% of total ApoA-I clearance. The residence times (RT) of ApoA-I in Tangier disease patients have been measured to be 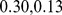 day in [74] and 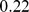 day in [75]. It is assumed that in Tangier patients, 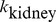 = 1/RT. Thus, 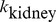 has been estimated to be 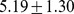 pool/day (mean±SEM). Because of the assumption made in mapping ApoA-I clearance measured in the Tangier patients to the normal population, we apply the factor 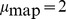 to give 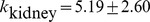 pool/day (mean±SEM).

#### Dissociation rate constant of labile ApoA-I (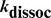)

In [76], fluorescence resonance energy transfer spectroscopy was used to quantify the rate of ApoA-I exchange. In this *in-vitro* set-up using synthetic rHDL incubated with 5-molar excess of lipid-free ApoA-I, the exponential relaxation time (defined as the time by which 50% of the exchange has occurred) was inferred to be 0.94 hour. This gives rise to the estimate of 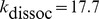 pool/day [76]. The ApoA-I found on *α*-HDL particles can be divided into a tightly-bound pool [77] and a labile pool [78]. The study of the dissociation of ApoA-I molecules from the labile pool is carried out in [78], where surface plasmon resonance was used to study the kinetics of ApoA-I interaction with HDL particles. A two-state binding model was used to describe the association and dissociation reactions and the rate parameters were identified from the time-course data. It has been found that for the pool of ApoA-I molecules that are bound to HDL particles in a stable conformation, the half-time of dissociation is around 3 minutes, corresponding to 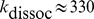 pool/day. Computing the mean and SD of the two estimates of 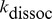 and using 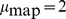 to account for the fact that these values were measured *in-vitro*, we obtain 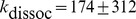 pool/day (mean±SEM).

#### Rate constant of the lipidation of lipid-poor ApoA-I via ABCA1 (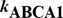)

The model assumes that the lipidation of ApoA-I is initiated by ABCA1, leading to the formation of nascent discs and subsequently to nascent spheres (via LCAT). While LCAT is crucial for the esterification of free cholesterol to cholesteryl ester, ABCA1 activity is assumed to be rate-limiting in the formation of *α*-HDL. In the model, the rate at which the concatenation of processes leading from lipid-poor ApoA-I to mature, *α*-HDL is described by the ABCA1 activity, 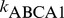. Based on the size exclusion chromatographic technique for separating HDL into subclasses, the rate constant in the conversion of lipid-poor ApoA-I to the *α*-HDL pool has been estimated to be 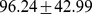 pool/day (mean±SD, n = 6) [22]. Thus, the mean and SEM is given by 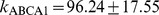 pool/day (mean±SEM).

#### Stoichiometry of FC to ApoA-I in nascent discs (*γ*)

In the model, *γ* denotes the stoichiometry (or molar ratio) of FC to ApoA-I in nascent discs. Due to the model assumption that FC in nascent discs are all esterified and result in the formation of nascent spheres, *γ* also equals the stoichiometry of CE to ApoA-I entering the *α*-HDL pool. Given these two interpretations of the model parameter *γ*, there are alternative ways to estimate it from literature data. In [79], plasma was fractionated using two-dimensional electrophoresis and the composition of pre-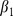 was analyzed. The weight fraction of FC to ApoA-I was found to be 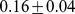 (n = 4). Using the given molecular weights of ApoA-I and FC, we obtain the molar ratio of FC/ApoA-I = 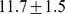 (mean±SEM). An alternative estimate of *γ* is obtained using the estimates for the rate of cholesterol esterification to HDL-CE (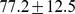 mg/dL/day, *n* = 3) [20] as well as the production rate of *α*-HDL from pre-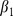 (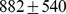 mg/dL/day, *n* = 6) [22]. Thus, using these sets of data *γ* is estimated to be 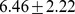 (mean ± SEM). Finally, an *in-vitro* experiment has been carried out to characterize the composition of nascent HDL (nHDL) formed by the action of ABCA1 on ApoA-I [80]. For the small nHDL formed (diameter ≈7.5 nm), the particles were found to contain, on average, 2 ApoA-I and 9 total cholesterol. This gives the estimate for *γ* of 4.5. Thus, combining all three estimates we get 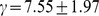 (mean±SEM). Using the factor 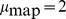 to take into account that *in-vitro* estimates were used, we obtain the final estimate of 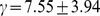 (mean±SEM).

#### Rate constant of CE transfer from HDL to VLDL (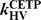)

In [20], the unidirectional movement of CE from HDL to VLDL was quantified using a two -pool model for CE in the Apo B-100 particle classes (VLDL and LDL) and a single pool for CE in HDL particles. Using the data from 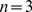 subjects, 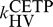 is estimated to be 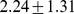 pool/day (mean±SD). The rate of CE movement from HDL particles to VLDL has also been quantified in [21]. In this compartmental analysis, a 3-pool model has been used for CE in Apo-B particles (VLDL, IDL and LDL) and bidirectionality of transfer has been assumed between HDL and VLDL as well as between HDL and LDL. Due to the fact that our model does not account for IDL, the CE transfers for this density class are pooled into those of VLDL. For 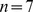 normal subjects, the net transfers of CE from HDL to VLDL and IDL are 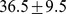 mg/dL/day. Normalizing by the individual concentrations of HDL-CE, this gives the estimate of 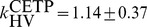 pool/day (mean±SD). By pooling the data sets from both Ouguerram *et al* [20] and Schwartz *et al* [21] and using 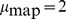 to account for the difference in the structures of compartmental models, we obtain: 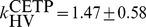 pool/day (mean±SEM).

#### Rate constant of CE transfer from HDL to LDL (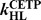)

In [20], the movement of CE from HDL to LDL was quantified using a two -pool model for CE in the Apo B-100 particle classes (VLDL and LDL) and a single pool for CE in HDL particles. Using the data from the 3 subjects, 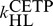 is estimated to be 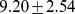 pool/day (mean±SD). A different estimate is obtained using the data for 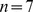 normal subjects given in [21]: by dividing the rates of CE movement from HDL particles to LDL by the concentrations of HDL-CE at the individual level, 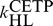 is estimated to be 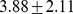 pool/day (mean±SD). By pooling the data sets from both Ouguerram *et al* [20] and Schwartz *et al* [21] and using 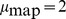 to account for the difference in the structures of compartmental models, we obtain: 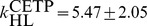 pool/day (mean±SEM).

#### Rate constant of CE transfer from LDL to HDL (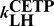)

In [20], the rate of CE transfer from LDL to HDL was quantified using a two -pool model for CE in the Apo B-100 particle classes (VLDL and LDL) and a single pool for CE in HDL particles. Using the data from the 3 subjects, 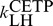 is estimated to be 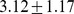 pool/day (mean±SD). An alternative estimate is obtained using the data for 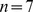 normal subjects given in [21]: by dividing the rates of CE movement from LDL particles to HDL by the concentrations of LDL-CE at the individual level, 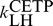 is estimated to be 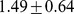 pool/day (mean±SD). By pooling the data sets from both Ouguerram *et al* [20] and Schwartz *et al* [21] and using 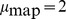 to account for the difference in the structures of compartmental models, we obtain: 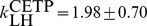 pool/day (mean±SEM).

#### Rate constant of transfer of CE from VLDL to LDL (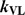)

In [20], the rate constant of CE transfer from VLDL to LDL (due primarily to lipolysis) was inferred in 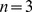 normal subjects: this gives rise to the parameter estimate 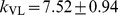 pool/day (mean±SEM).

#### Flux of CE to VLDL (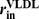)

In [20], the flux of CE into the VLDL pool was inferred in 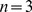 normal subjects to be 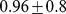 mg/dL/day (mean±SD). The cholesteryl ester production to VLDL was also measured by Schwartz *et al* in [21], but due to the large uncertainty as represented by the greater than 100% SD in some of the individual data, these values have not been used. Thus, we take 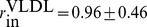 mg/dL/day (mean±SEM).

#### Rate constant of CE elimination from VLDL (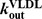)

In [20], the rate constant of CE elimination from the VLDL pool was inferred in 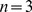 normal subjects to be 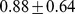 pool/day (mean±SD). The quantification of CE elimination rate from VLDL was also carried out by Schwartz *et al* in [21], but the mean of the data was not shown in the paper because most values were undefined (fractional SD >80%). Thus, we use only the values given by Ouguerram *et al* [20] and take 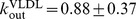 pool/day (mean±SEM).

#### Rate constant of CE elimination from LDL (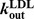)

In [20], the rate constant of CE elimination from the LDL pool was inferred in 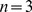 normal subjects to be 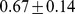 pool/day (mean±SD). The quantification of CE elimination rate from LDL was also carried out by Schwartz *et al* in [21], but the mean of the flux to the extra-hepatic pool was not shown in the paper because most values were undefined (fractional SD >80%). Thus, we use only the value given by Ouguerram *et al* [20] and take 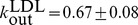 pool/day (mean±SEM).

#### Rate constant of SRB1-mediated CE elimination from HDL (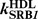)

In [20], the rate constant of selective CE elimination from the HDL pool was inferred in 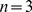 normal subjects to be 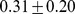 pool/day (mean±SD). The selective uptake of HDL-CE by the liver was not observed in Schwartz *et al* in [21]. Hence, we use only the value given by Ouguerram *et al* [20] and take 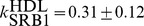 pool/day 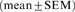.

#### Rate constant of size-independent holo-particle uptake for *α*-HDL (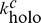)

The FCR of ApoA-I in the *α*-HDL pool has been estimated to be 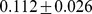 pool/day (mean±SD, n = 6) using HDL subclasses separated with size exclusion chromatography [22]. In another reference [72], using a separation technique based on agarose gel electrophoresis, the FCR of ApoA-I in the *α*-HDL pool has been estimated to be 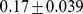 pool/day (mean±SD, *n* = 6). Thus, by pooling the data we obtain 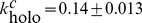 pool/day (mean±SEM). Finally, using the factor 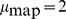, we get 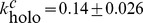 pool/day (mean±SEM).

### Calibration data

In this section, we give the quantitative values and references for the data used in the calibration procedure.

#### CETP mutation

Lipoprotein data for CETP mutation patients were taken from 3 sources: Inazu *et al* [81], Yamashita *et al* [82] and Asztalos *et al* [83] and were pooled to yield the mean and SEM. In particular, both HDL-C and ApoA-I were available for each of the 3 data sources shown in Table 6. The values for LDL-CE and VLDL-CE for the control subjects were not given in the references for CETP mutation [81]–[83]; hence, the values from Ouguerram *et al* [20] and Schwartz *et al* [21] were used in Table 7. The value of LDL-CE for CETP heterozygotes was also used for calibrating the model, which was estimated using the given value of LDL-C with an assumption on the ratio of FC/CE for LDL particles. In Asztalos *et al* [83], the ratio of FC/CE for all lipoprotein particle classes of CETP heterozygotes was given as 0.37, which gives CE = 0.73×TC. Given the approximations made, we assumed that LDL-CE = LDL-C 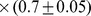. There is literature data for LDL-CE and/or VLDL-CE in CETP homozygotes [39]–[41], but these were not used in the calibration process due to the known inconsistency of the model in lacking the *β*-LCAT activity [42].

**Table 6:** Calibration data: HDL-C and ApoA-I in normal and CETP deficient subjects.

Type	Data	Inazu [81] (mean±SD)	Yamashita [82] (mean±SD)	Asztalos [83] (mean±SD)	Pooled (mean±SEM)
Normal Subjects	*n*	16	20	50	86
	HDL-C	52.9±13.9	50±8	52±14	52±1
	ApoA-I	124±21	140.9±16.1	144±29	139±4
Heterozygotes of CETP deficiency	*n*	20	15	5	40
	HDL-C	66±15	84±25	85±26	75±4
	ApoA-I	149±43	155.3±22.1	154±25	152±5
Homozygotes of CETP deficiency	*n*	10	4	9	23
	HDL-C	163.7±39	193±28	157±29	166±7
	ApoA-I	213±47	233.5±22.3	252±25	232±8

All concentrations are given in mg/dL.

**Table 7:** Calibration data: CE in ApoB particles.

Type	Data [20], [21]	Pooled (mean±SEM)
Normal subjects	*n*	10
	LDL-CE	83±3 mg/dL
	VLDL-CE	5±1 mg/dL
Heterozygotes of CETP deficiency	*n*	23
	LDL-CE (LDL-C ×0.7)	72±9 mg/dL

#### Cholesteryl ester flux

There are 2 literature data sources on the *in-vivo* flux of CE between HDL and ApoB-containing particles: Ouguerram *et al* [20] and Schwartz *et al* [21]. While both data sets have been used in estimating the prior distribution of parameters involved in the exchanges of CE (see Methods section), for the purpose of model calibration a choice between the 2 disparate data needed to be made. Given that the model structure in the description of CE exchanges between HDL, LDL and VLDL is based on that of Ouguerram *et al* [20], it was decided that the same calibration data should be taken for the CE fluxes. The values used are shown in Table 8.

**Table 8:** Calibration data: CE fluxes.

CE flux (mean±SEM)	Data (mg/dL/day)
HDL to VLDL	64±2
HDL to LDL	268±51
LDL to HDL	266±52

#### Fractional catabolic rate of apoA-I

Brinton *et al* have shown that a strong association exists between the fractional catabolic rate (FCR) of ApoA-I and the estimated HDL size, using a surrogate marker [43] (Brinton *et al* used HDL-C/(ApoA-I+ApoA-II), we re-analyzed their data taking HDL-C/ApoA-I as the surrogate marker). Their work has demonstrated that as much as 70% of the variability in the FCR of ApoA-I may be attributed to variations in HDL size, as estimated using 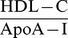 [43]. This finding is corroborated with the individual data of ApoA-I metabolism from Schaefer *et al* [44] measured in healthy volunteers. Finally, FCR of ApoA-I was also studied by Ikewaki *et al* [45] in comparing CETP mutation subjects with controls. All these data consistently show that the ApoA-I FCR exhibits an inverse relationship with the surrogate measure of HDL size. However, the trend does not continue indefinitely: even for CETP homozygous subjects with very large particles, ApoA-I FCR appears to reach a minimum of 0.135 pool/day. Hence, we have tried to describe the data in the simplest way, assuming a linear relationship between ApoA-I FCR and 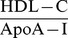, with a lower bound of 0.135. Using this linear assumption, the fit and confidence interval was computed and shown in Figure 4. The piecewise linear fit to the combined data is given by 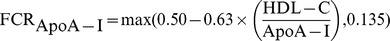; the mean confidence interval corresponding to 1 SD in the line fit is approximately ±0.065 pool/day.

### Model calibration

In this work, we assume that both the parameter prior and the data error are normally distributed. We employ the methodology of *maximum a posteriori* (MAP) [31] to combine the prior information with calibration data. Due to the conjugacy property [31] of the distributions, the posterior also has a normal distribution and the MAP solution is obtained by solving a nonlinear least squares problem. In our model, most of the parameters have an informative prior. For the set of parameters for which an informative prior is available, let 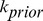 denote the expected value of the prior distribution; otherwise, set 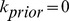 to represent the lack of information. We take the covariance matrix for the prior distribution 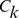 to have a diagonal structure: for parameters 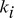 that have an informative prior, 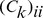 is the variance of the prior distribution; for parameters that have an uninformative prior, 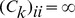. That is, the prior distribution is assumed to be of the form [33]:
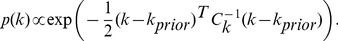
(16)Let 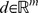 denote the vector of calibration data and 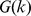 the nonlinear mapping from model parameters to the observation, representing the model simulation of the data. Let 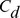 denote the covariance matrix for the data. Hence, the conditional distribution of the data given the model parameter *k* is [33]:
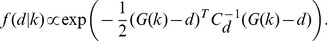
(17)Thus, the posterior distribution 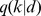 for the model parameters is given by
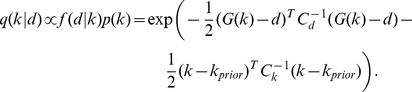
(18)To find the MAP solution, the following nonlinear least squares problem is solved: with the objective function defined as,
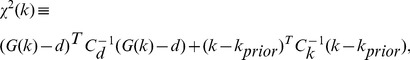
(19)the MAP solution is the minimizer:
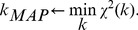
(20)Using parameter priors as given in Table 5 and calibration data as described in the previous section, the nonlinear least-squares problem was solved using genetic algorithm ga from the Matlab® Global Optimization Toolbox of MathWorks (http://www.mathworks.com/) to obtain 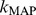. In particular, the hybrid option was selected: 100 generations of the genetic algorithm was run with a PopulationSize = 500, followed by constrained minimization (fmincon) using the setting MaxFunEvals = 10000, MaxIter = 1000. In all numerical integration of ODEs, the relative and absolute tolerances were set to 10^−9^.

### Estimation of 95% confidence intervals

The confidence interval is estimated using the following procedure: parameters are sampled around 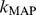 and for each parameter the 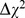 (with respect to its minimum, 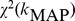) is computed according to the expression (19). An estimate of the confidence region is obtained by examining the set of all parameters that lie within 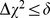, where *δ* is computed from the number of degrees of freedom (df) and the desired confidence level [84]. Using df = 29 for the model and choosing the 95% confidence level, 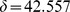. A set of 1000 parameters satisfying 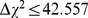 are selected in estimating confidence intervals shown in the paper.

### Simulation of tracer kinetic studies

The model simulations of the tracer kinetic experiment with labelled ApoA-I and the calculation of the FCR of ApoA-I were carried out using the technique of complex variable differentiation [85]. In particular, a small quantity of imaginary number representing the radio-labelled dose of ApoA-I is added to the lipid-poor pool at the start of tracer experiment and the imaginary component of the numerical solution is extracted to represent the dose remaining in the two pools of ApoA-I (lipid-poor and *α*-HDL). This method relies on the complex extension of analytic functions from the real line, which can be easily implemented on the Matlab platform [85]. As compared to the finite-differencing approach, the complex variable methodology does not suffer from subtractive cancellation error and hence is more accurate [85]. While this approach has not been applied to tracer kinetic simulations, it has been applied to the sensitivity analysis of biological models [86], [87].

## Supporting Information

File S1 **LMK model implementation in SimBiology and Matlab formats.**
(ZIP)
