# Correction: An *In-Silico* Model of Lipoprotein Metabolism and Kinetics for the Evaluation of Targets and Biomarkers in the Reverse Cholesterol Transport Pathway

**DOI:** 10.1371/journal.pcbi.1004001

**Published:** 2014-11-04

**Authors:** The PLOS Computational Biology Staff

The parameter k_f in Table 5 is incorrect. It should be given as 50000. Correspondingly the standard deviation for the parameter k_f should also be ±15440.
